# A Broad Spectrum Protein Glycosylation System Influences Type II Protein Secretion and Associated Phenotypes in *Vibrio cholerae*

**DOI:** 10.3389/fmicb.2019.02780

**Published:** 2019-12-03

**Authors:** Dina Vorkapic, Fabian Mitterer, Katharina Pressler, Deborah R. Leitner, Jan Haug Anonsen, Laura Liesinger, Lisa-Maria Mauerhofer, Torben Kuehnast, Manuela Toeglhofer, Adina Schulze, Franz G. Zingl, Mario F. Feldman, Joachim Reidl, Ruth Birner-Gruenberger, Michael Koomey, Stefan Schild

**Affiliations:** ^1^Institute of Molecular Biosciences, University of Graz, Graz, Austria; ^2^Centre for Ecological and Evolutionary Synthesis, Department of Biosciences, University of Oslo, Oslo, Norway; ^3^Institute of Pathology, Medical University of Graz, Graz, Austria; ^4^Omics Center Graz, BioTechMed-Graz, Graz, Austria; ^5^Department of Molecular Microbiology, Washington University School of Medicine in St. Louis, St. Louis, MO, United States; ^6^BioTechMed-Graz, Graz, Austria; ^7^Institute of Chemical Technologies and Analytics, Vienna University of Technology, Vienna, Austria

**Keywords:** post-translational modification, *O*-OTase, *Vibrio cholerae*, biofilm, virulence, chaperone

## Abstract

Protein secretion plays a crucial role for bacterial pathogens, exemplified by facultative human-pathogen *Vibrio cholerae*, which secretes various proteinaceous effectors at different stages of its lifecycle. Accordingly, the identification of factors impacting on protein secretion is important to understand the bacterial pathophysiology. PglL_Vc_, a predicted oligosaccharyltransferase of *V. cholerae*, has been recently shown to exhibit *O*-glycosylation activity with relaxed glycan specificity in an engineered *Escherichia coli* system. By engineering *V. cholerae* strains to express a defined, undecaprenyl diphosphate-linked glycoform precursor, we confirmed functional *O*-linked protein glycosylation activity of PglL_Vc_ in *V. cholerae*. We demonstrate that PglL_Vc_ is required for the glycosylation of multiple *V. cholerae* proteins, including periplasmic chaperones such as DegP, that are required for efficient type II-dependent secretion. Moreover, defined deletion mutants and complementation strains provided first insights into the physiological role of *O*-linked protein glycosylation in *V. cholerae*. RbmD, a protein with structural similarities to PglL_Vc_ and other established oligosaccharyltransferases (OTases), was also included in this phenotypical characterization. Remarkably, presence or absence of PglL_Vc_ and RbmD impacts the secretion of proteins via the type II secretion system (T2SS). This is highlighted by altered cholera toxin (CT) secretion, chitin utilization and biofilm formation observed in Δ*pglL*_Vc_ and Δ*rbmD* single or double mutants. This work thus establishes a unique connection between broad spectrum *O*-linked protein glycosylation and the efficacy of type II-dependent protein secretion critical to the pathogen’s lifecycle.

## Introduction

The Gram-negative pathogen *Vibrio cholerae*, the causative agent of the severe secretory diarrheal disease cholera, transits between two dissimilar habitats along its life cycle. *V. cholerae* efficiently colonizes the human gastrointestinal tract upon oral ingestion of contaminated food or water, but also persists as a natural inhabitant in aquatic ecosystems during inter-epidemic periods (Schild et al., 2008; Nelson et al., 2009). *V. cholerae* employs a number of strategies to quickly adapt to the differential conditions faced along its life cycle, which ensures survival even under unfavorable conditions.

A hallmark of environmental survival and transmission of *V. cholerae* is the ability to form biofilms on chitinous surfaces in the aquatic reservoirs. Ubiquitous chitin particles in the nutrient-limited aquatic reservoir not only act as an attachment platform for subsequent biofilm formation, but also serve as a carbon and nitrogen source. Accordingly, *V. cholerae* expresses a complex chitin utilization program including several secreted chitinases (Meibom et al., 2004). Once ingested by humans, *V. cholerae* passes the acidic barrier in the stomach and reaches the small intestine, the primary site of colonization. During this passage, *V. cholerae* activates a complex regulatory cascade to induce the expression of virulence factors and achieve full colonization fitness (Childers and Klose, 2007). The pathology of cholera is mainly due to the activity of the secreted cholera toxin (CT), which induces a massive efflux of chloride ions and water into the intestinal lumen resulting in a secretory diarrhea (Childers and Klose, 2007).

Several proteins crucial for survival fitness along the different stages of the life cycle are secreted via the type II secretion system (T2SS). These include CT, the biofilm matrix proteins (RbmA, RbmC, and Bap1), and chitinases along with complementary proteins involved in chitin metabolism (Sikora et al., 2011; Johnson et al., 2014). Adaptation to different conditions has so far been mainly investigated through transcriptional profiling, although there is growing evidence that *V. cholerae* also takes advantage of post-translational regulatory strategies. These are exemplified by proteolysis control of transcription factors TcpP and ToxR affecting virulence factor expression, RpoS responsible for the mucosal escape response and FliA, which inversely controls flagella gene transcription and virulence gene expression (Matson and DiRita, 2005; Nielsen et al., 2006; Almagro-Moreno et al., 2015; Pressler et al., 2016; Rogers et al., 2016; Wurm et al., 2017).

*O*-linked protein glycosylation appears to be widely distributed in bacteria and represents a post-translational modification whereby oligosaccharyltransferases (OTases) transfer a pre-assembled glycan onto target proteins (Iwashkiw et al., 2013). Based on current models for Gram-negative bacteria, the glycan is synthesized on an undecaprenyl pyrophosphate (Und-PP) lipid carrier in the cytoplasm, flipped across the inner membrane and utilized by *O*-OTases to glycosylate serine or threonine residues of target proteins in the periplasm (Nothaft and Szymanski, 2010). Probably the best studied *O*-glycosylation system of Gram-negative bacteria is the protein glycosylation (*pgl*) in *Neisseria gonorrhoeae*. Briefly, PglB, PglC, and PglD are required for synthesis of Und-PP linked di-*N*-acetyl bacillosamine (diNAcBac), the proximal sugar in the oligosaccharide (Aas et al., 2007; Hartley et al., 2011). The fully synthesized glycan is flipped to the periplasmic space by the flippase PglF and subsequently transferred by the OTase PglL/O on diverse target proteins via en block transfer (Aas et al., 2007; Vik et al., 2009).

Intrigued by a potential, intrinsic *V. cholerae* post-translational modification system (Gebhart et al., 2012), we constructed and characterized a *pglL*_Vc_ null mutant. Using a reverse glycoengineering strategy, we found that PglL_Vc_ is required for the glycosylation of multiple *V. cholerae* proteins. In addition, the *pglL*_Vc_ null mutant showed altered biofilm features and a trend toward enhanced type II substrate secretion. Remarkably, these phenotypes were significantly augmented in a background simultaneously lacking RbmD, a protein of unknown function with limited structural similarities to PglL_Vc_ and other established oligosaccharyltransferases (Supplementary Figure S1). Thus, this work reveals genetic interactions between *pglL*_Vc_ and *rbmD*, and establishes clear connections between broad spectrum *O*-linked protein glycosylation and secretion processes important in diverse pathogen life styles.

## Materials and Methods

### Strain Construction and Growth Conditions

Bacterial strains and plasmids or oligonucleotides used in this study are listed in Tables 1, 2, respectively. The clinical isolate *V. cholerae* O1 El Tor C6709 was used as WT strain in all experiments. *Escherichia coli* strains DH5αλpir and SM10λpir were used for genetic manipulations. Unless stated otherwise, all strains were grown with aeration in lysogeny broth (LB, 1% tryptone; 1% NaCl; 0.5% yeast extract) on 37°C, on LB agar plates at 37°C, or for biofilm formation under static conditions at 24°C. To assess CT expression and secretion, *V. cholerae* strains were grown under virulence gene inducing conditions at 37°C for 4 h anaerobically using AKI broth, followed by 4 h aerobic growth with shaking at 180 rpm (Iwanaga and Yamamoto, 1985; Iwanaga et al., 1986). Minimal media M9 was prepared according to standard recipe (Miller, 1972), and is indicated as M9-*X*, whereby *X* represents the sole carbon source used. Antibiotics and other supplements were used in the following final concentrations: streptomycin (Sm, 100 μg/ml), ampicillin (Ap, 50 μg/ml in combination with other antibiotics, otherwise 100 μg/ml), kanamycin (Km, 50 μg/ml), chloramphenicol (Cm, 20 μg/ml for *E. coli* strains or 2 μg/ml for *V. cholerae* strains), sucrose (10%), glucose (Glc, 0.2%), chitin (0,2%, Sigma-Aldrich), *N*-acetylglucosamine (GlcNAc, 0.2%).

**TABLE 1:** Strains and plasmids used in this study.

	**Strain description**	**References**
***E. coli***		
DH5Δλpir	F-ϕ(*lacZYA*-*argF*) *U169recA1endA1 hsdR17 supE44 thi-1 gyrA96 relA1*λ:*pir*	Kolter et al., 1978
SM10λpir	*thi recA thr leu tonA lacY supE RP4-2-Tc:Mu*λ*:pir*	Miller and Mekalanos, 1988
CLM24	Constructed from *E. coli* W3110 (IN(rrnD-rrnE)1 rph-1). *waaL* mutant	Feldman et al., 2005
***V. cholerae***		
WT	C6709, O1 El Tor Inaba, clinical isolate, 1991 Peru, *tcpA*^+^ *ctxAB*^+^ *hapR*^+^, spontaneous Sm^r^	Roberts et al., 1992
C6709*lacZ*	C6709, *lacZ*:res-*neo-sacB*-res	Tamayo et al., 2008
Δ*pglL*_Vc_	Deletion of *pglL*_Vc_ (VC0393) in C6709, Sm^r^	This work
Δ*rbmD*	Deletion of *rbmD* (VC0931) in C6709, Sm^r^	This work
Δ*rbmA*	Deletion of *rbmA* (VC0928) in C6709, Sm^r^	This work
Δ*pglL*_Vc_Δ*rbmD*	Deletion of *pglL*_Vc_ and *rbmD* in C6709, Sm^r^	This work
Δ*pglL*_Vc_Δ*rbmD*Δ*rbmA*	Deletion of *pglL*_Vc_, *rbmD* and *rbmA* in C6709, Sm^r^	This work
Δ*degP:cat*	Deletion of *degP* (VC0566) in C6709, Sm^r^, Cm^r^	This work
Δ*pglL*_Vc_Δ*rbmD* Δ*degP:cat*	Deletion of *pglL*_Vc_, *rbmD* and *degP* in C6709, Sm^r^, Cm^r^	This work
Δ*epsC-N*	Deletion of *epsC-N* (VC2734-VC2723) in C6709, Sm^r^	This work
Δ*hapR*	Deletion of *hapR* in C6709, Sm^r^	Seper et al., 2011
*vpsA*:*phoA*	Insertion of pGP*phoA* in *vpsA* of C6709, Sm^r^, Ap^r^	Seper et al., 2011
Δ*hapRvpsA*:*phoA*	Insertion of pGP*phoA* in *vpsA* of Δ*hapR*, Sm^r^, Ap^r^	Seper et al., 2011
Δ*pglL*_Vc_Δ*rbmD vpsA*:*phoA*	Insertion of pGPphoA in *vpsA* of 9Δ*pglL*_Vc_Δ*rbmD*, Sm^r^, Ap^r^	This work
Δ*lacZ*:*gfp*	Insertion of *gfp* in the *lacZ* of C6709, Sm^r^	This work
Δ*pglL*_Vc_Δ*lacZ*:*gfp*	Insertion of *gfp* in the *lacZ* of Δ*pglL*_Vc_, Sm^r^	This work
Δ*rbmD*Δ*lacZ*:*gfp*	Insertion of *gfp* in the *lacZ* of Δ*rbmD*, Sm^r^	This work
Δ*pglL*_Vc_Δ*rbmD*Δ*lacZ*:*gfp*	Insertion of *gfp* in the *lacZ* of Δ*pglL*_Vc_Δ*rbmD*, Sm^r^	This work
Δ*vps*-I:Km	Deletion of *vps*-I cluster (VC0917-VC0927) in C6709 by exchange with a kanamycin cassette, Sm^r^, Km^r^	This work
Δ*vps*-I	Deletion of kanamycin cassette in Δ*vps*-I:Km, Sm^r^	This work
Δ*pglL*_Vc_Δ*rbmD*Δ*vps*-I:Km	Deletion of *vps*-I cluster (VC0917-VC0927) in Δ*pglL*_Vc_Δ*rbmD* by exchange with a kanamycin cassette, Sm^r^, Km^r^	This work
Δ*pglL*_Vc_Δ*rbmD*Δ*vps*-I	Deletion of kanamycin cassette in Δ*pglL*_Vc_Δ*rbmD*Δ*vps*-I:Km, Sm^r^	This work
Δ*rfb*:Km	Deletion of *rfbA* to *rfbU* (VC0241-VC0260) in C6709 by exchange with a kanamycin cassette, Sm^r^, Km^r^	This work
Δ*rfb*	Deletion of kanamycin cassette in Δ*rfb*:Km, Sm^r^	This work
**Plasmids**		
pCVD442	*ori6K mobRP4 sacB*, Ap^r^	Donnenberg and Kaper, 1991
pJZ111	*Plac*:*gfp*:*lacZ* in pCVD442, Ap^r^	Seper et al., 2014
p	pMMB67EH, IncQ broad-host-range low-copy-number cloning vector, IPTG inducible, Ap^r^	Morales et al., 1991
pMMBneo	pMMB67EH-based plasmid, IncQ broad-host-range low-copy-number cloning vector, IPTG inducible, Kan^r^	Tamayo et al., 2008
pQE60	C-terminal His-tag expression plasmid, Ap^r^	Qiagen
pAC1000	Cm^r^	Hava et al., 2003
pACYC184	Cloning and expression vector, p15A ori, IPTG inducible, derived from pACT3, Cm^r^	Chang and Cohen, 1978
pACYC*pglFBCD*	pACYC184-based plasmid containing the *pglFBCD* locus from *N. gonorrhoeae*, Cm^r^	Egge-Jacobsen et al., 2011
pGPphoAvpsA	pGPphoA with “*vpsA*” fragment of C6709, Ap^r^	Seper et al., 2011
pCVDΔ*pglL*_Vc_	pCVD442 with up- and downstream fragments of *pglL*_Vc_, Ap^r^	This work
pCVDΔ*rbmD*	pCVD442 with up- and downstream fragments of *rbmD*, Ap^r^	This work
pCVDΔ*rbmA*	pCVD442 with up- and downstream fragments of *rbmA*, Ap^r^	This work
pCVDΔ*degP:cat*	pCVD442 with up- and downstream fragments of *degP* flanking a cat cassette, Ap^r^, Cm^r^	This work
pCVDΔ*epsC-N:cat*	pCVD442 with upstream fragment of *epsC* and downstream fragment of *epsN* flanking a cat cassette, Ap^r^, Cm^r^	This work
p*-*pglL_Vc_	*pglL*_Vc_ of *V. cholerae* in pMMB, Ap^r^	This work
p-rbmD	*rbmD* of *V. cholerae* in pMMB, Ap^r^	This work
pMMBneo-pglL_Vc_	*pglL*_Vc_ of *V. cholerae* in pMMBneo, Km^r^	This work
pMMBneo-rbmD	*rbmD* of *V. cholerae* in pMMBneo, Km^r^	This work
pMMB-degP	*degP* of *V. cholerae* in pMMB, Ap^r^	
pQE60-rbmA	*rbmA* of *V. cholerae* in pQE60, Ap^r^	This work
pQE60-degP	*degP* of *V. cholerae* in pQE60, Ap^r^	This work
pCVDΔ*vpsA*:KanI	pCVD442 with upstream and downstream fragment of *vpsA* flanking 2/3 of a kanamycin resistance cassette, Ap^r^, Km^r^	This work
pGP*vpsK*:KanII	pGP704 with a fragment of *vpsK* flanking 2/3 of a kanamycin resistance cassette, Ap^r^, Km^r^	This work
pCVDΔ*vpsA*-*K*	pCVD442 with an upstream fragment of *vpsA* and a downstream fragment of *vpsK*, Ap^r^, Km^r^	This work
pCVDΔ*rfbA*:KanI	pCVD442 with upstream and downstream fragment of *rfbA* flanking 2/3 of a kanamycin resistance cassette, Ap^r^, Km^r^	Schild et al., 2005
pGP*rfbU*:KanII	pGP704 with a fragment of *rfbU* flanking 2/3 of a kanamycin resistance cassette, Ap^r^, Km^r^	This work
pCVDΔ*rfbA*-*U*	pCVD442 with an upstream fragment of *rfbA* and a downstream fragment of *rfbU*, Ap^r^, Km^r^	This work
		

**TABLE 2:** Oligonucleotides used in this study.

**Primer name**	**Sequence (5′–3′)^*a*^**
VC0393_*Xba*I_1	AGGTCTAGAACACTTTGATA
VC0393_EcoRI_2	AAAGAATTCCACCTTAAAAAAGAAACCA
VC0393_EcoRI_3	AATGAATTCTAAGCTCTGCGAGAAACA
VC0393_*Sac*I_4	AATGAGCTCTAGCGAAGCAGCATGG
VC0931_*Sac*I_1	AAGAGCTCCAAATTATTTAACAAGCCACA
VC0931_*Bam*HI_2	AAGGATCCCATAAATTCGGTGGCTCAA
VC0931_*Bam*HI_3	TTGGATCCTGATTGCTCTTCATCTCC
VC0931_*Xba*I_4	TTTCTAGACACGGCAACACTTTGGG
VC0928_*Xba*I_1	TTTTCTAGAGCTAAGTTGGCTAAT
VC0928_EcoRI_2	TTTGAATTCCAACCATTTGTTTTTACAACT
VC0928_EcoRI_3	TTTGAATTCTAAATTTACCTAGTCACTTAG
VC0928_*Sac*I_4	TTAGAGCTCACTACAACACAAGCGACA
VC0566_Xbal_1	TTATCTAGAAGGTCGTACCACTGGCT
VC0566_EcoRI_2	AAAGAATTCATAAGCTCCTCAACAAATTAA
VC0566_EcoRI_3	AATGAATTCTAATTATCACTCAGCGTTACC
VC0566_*Sac*I_4	TTTGAGCTCGGTGGCTTGTTGAAAAGA
VC0393_*Sac*I_pMMB_fw	TTGAGCTCGAACTGTGCGCTGGTTTC
VC0393_SphI_pMMB_rev	TTGCATGCCACCCTTATTGTTTCTCG
VC0931_EcoRI_pMMB_fw	TTGAATTCCTATTTTGAGCCACCGAA
VC0931_*Bam*HI_pMMB_rev	TTGGATCCAGTGATCATGCAGGAGAT
VC0928_*Nco*I_pQE60_fw	AAACCATGGACAAACGTCATTATTATCTGG
VC0928_BglIII_pQE60_rev	TTTAGATCTTTTTTTTACCACTGTCATTGA
VC0566_*Nco*I_pQE60_fw	TAACCATGGAAAAACCTTTACTTGTTTTAACT
VC0566_*Bam*HI_pQE60_rev	TTAGGATCCACGAACAACCAAGTAAAGCGT
VC2734_*Sac*I_1	AAAGAGCTCGCCTTGCTTAGGTTCA
VC2734_*Bam*HI_2	TTAGGATCCCATAAATTTCCACGTTATTCC
VC2723_EcoRI_3	AATGAATTCTAGGATGTGTAATCCCATTCA
VC2373_*Xba*I_4	TTTTCTAGATCATTCGCTGGCCTTTA
cat_EcoRI_fw	TTTGAATTCGATAAGCTTGATGAAAATTTG
cat_EcoRI_rev	TTAGAATTCAGGTTAGTGACATTAGAAAA
vpsA_*Sac*I_1	TTGAGCTCTAGTCGTGTGTACAGGTAATT
vpsA_*Bam*HI_2	TTCCTAGGCACTTCCCCACATCCTCT
vpsA_EcoRI_3-1	TTGAATCCTACTTCCCGAGTAGGCACAA
vpsA_*Xba*I 4-1	ATTCTAGAGAAACCAACTCGATA
vpsA_*Nco*I	TTCCATGGCACTTCCCCACATCCTCT
vpsK_*Nco*I	AATCCATGGTATGGGGGTGGTTT
vpsK_*Xba*I	AAATCTAGAAAAATACATCCGCTTTTATT
rfbA_*Sac*I	TTGAGCTCGATGTTTATACCTGTAATTATGGCT
rfbA_*Bam*HI	TAGGATCCACTGGCTAAATACTGCTCAGCAA
rfbU_*Bam*HI	AAAGGATCCAAATAAAGAGAGAAAGTCTCC
rfbU_*Xba*I	AATCTAGAATGTCTTAGGCTCTTCAGGT
kanI_*Bam*HI	ATGGATCCTTCAACTCAGCAAAAGT
kanI_EcoRI	TAGAATTCCGACTCGTCCAACATCAATA
kanII_*Sac*I	TAGAGCTCATGGATGCGTATTTATATGGGT
kanII_*Bam*HI	TCGGATCCGTCAGCGTAATGCTCTGCCAGT
kanII_*Nco*I	TCCCATGGTCAGCGTAATGCTCTGCCAGT

*^*a*^Restriction sites are underlined.*

### DNA Manipulations, Construction of Suicide Plasmids, Reporter Fusions, Mutant Strains, and Expression Plasmids

Qiaquick^®^ Gel extraction and Qiaquick^®^ PCR Purification kits (Qiagen) were used for purifying PCR products and digested plasmid DNA. PCR reactions for subcloning were carried out using the Q5^®^ High-Fidelity DNA Polymerase (NEB), whereas Taq DNA Polymerase (NEB) was used for all other PCRs. Plasmid DNA was isolated using plasmid DNA purification kits (Qiagen).

Constructions of in frame deletion mutants were carried out as described by Donnenberg and Kaper (1991). 800 bp long DNA fragments flanking the gene of interest were amplified using the oligonucleotide pairs *x*_*y*_1 and *x*_*y*_2 or *x*_*y*_3 and *x*_*y*_4, where *x* represents the gene and *y* the restriction site/enzyme used (Table 2). For the construction of pCVDΔ*degP:cat* the *cat*-fragment was additionally amplified using pAC1000 as template and the oligonucleotide pairs cat_EcoRI_fw and cat_EcoRI_rev. After digestion with the appropriate restriction enzyme, indicated by the name of oligonucleotide, PCR fragments were ligated into pCVD442 restricted with similar restriction enzymes (NEB). Obtained corresponding knockout plasmids are listed in Table 1. Generated plasmids were first transformed into *E.coli* Sm10λpir and mobilized into *V.cholerae* via conjugation. Exconjugants were purified by Sm^r^ and Ap^r^ selection, followed by sucrose selection, as previously described (Donnenberg and Kaper, 1991). Correct chromosomal deletions were confirmed by PCR. GFP expressing strains were generated by insertion of *gfp* in the *lacZ* locus using the suicide plasmid pJZ111 (Seper et al., 2014).

For the construction of Δ*vps-*I deletion mutant a method by Schild et al. (2005) was used, which enabled us to remove a relatively large chromosomal gene cluster (VC0917-VC0927). Briefly, the first step was the construction of a *vpsA* mutant carrying an insertion of the *kanR’* gene (originating from plasmid pACYC177), consisting of about two-thirds of its gene length (*KanI*), and including the promoter and Shine-Dalgarno sequences. 800 bp long upstream and downstream sequences of *vpsA* were amplified by PCR using oligonucleotide pairs vpsA_*Sac*I_1 and vpsA_*Bam*HI_2 or vpsA_EcoRI_3 and vpsA_*Xba*I_4 (Table 2). The 800-bp PCR fragment of KanI was generated using the oligonucleotide pairs kanI_*Bam*HI and kanI_EcoRI (Schild et al., 2005). The three PCR fragments were digested with appropriate restriction enzymes (NEB) and ligated together into a *Sac*I/*Xba*I-digested pCVD442, resulting in the plasmid pCVDΔ*vpsA*:KanI. The plasmid pCVDΔ*vpsA*:KanI was mobilized into *V. cholerae* via conjugation, obtaining the mutant C6709Δ*vpsA*:KanI after sucrose selection. In the second step, the suicide plasmid pGP704 harboring downstream sequence of *vpsK* and the last two-thirds (C′ terminal) of the ′*kanR* gene (KanII) was constructed. The PCR fragments were obtained using the oligonucleotide pairs vpsK_*Nco*I and vpsK_*Xba*I for 800 bp downstream of *vpsK* or KanII_*Sac*I and KanII_*Nco*I for KanII (Schild et al., 2005) (Table 2), digested with Xba/*Nco*I or *Nco*I/*Sac*I, and ligated into a *Sac*I/*Xba*I-digested pGP704, resulting in pGP*vpsK:*KanII. After conjugation of pGP*vpsK:*KanII in C6709Δ*vpsA*:KanI, we obtained Ap^r^ colonies. Colony purification of some of these cells in absence of Ap and subsequent selection for Km^r^ resulted in Km^r^/Ap^*s*^ colonies. Deletion of kanamycin cassette was achieved by a pCVD442 suicide vector mutagenesis using pCVDΔ*vpsA-K*, which constructed by amplifying PCR fragments using the oligonucleotide pairs vpsA_*Sac*I_1 and vpsA_*Nco*I as well as vpsK_*Nco*I and vpsK_*Xba*I, digested with Xba/*Nco*I or *Nco*I/*Sac*I, and ligated into a *Sac*I/*Xba*I-digested pGP704, resulting in pGP*vpsK:*KanII. The correct chromosomal deletion of genes *vpsA-K* was confirmed by PCR.

The Δ*rfb* deletion mutant was generated as described above (see Δ*vps-*I deletion mutant construction for details) and comprises a deletion spanning from *rfbA* to *rfbU* (VC0241-VC0260). This required the pCVDΔ*rfbA*:KanI (Schild et al., 2005) and pGP*rfbU:*KanII (Table 2). The latter was constructed by amplifying PCR fragments using the oligonucleotide pairs rfbU_*Bam*HI and rfbU_*Xba*I for 800 bp downstream of *rfbU* or KanII_*Sac*I and KanII_*Bam*HI for KanII (Schild et al., 2005) (Table 2), digested with Xba/*Bam*HI or *Bam*HI/*Sac*I, and ligated into a *Sac*I/*Xba*I-digested pGP704, resulting in pGP*rfbU:*KanII. Deletion of kanamycin cassette was achieved by a pCVD442 suicide vector mutagenesis using pCVDΔ*rfbA-U*, which was constructed by amplifying PCR fragments using the oligonucleotide pairs rfbA_*Sac*I_1 and rfbA_*Bam*HI as well as rfbU_*Bam*HI and rfbU_*Xba*I, subsequent digestion with *Sac*I/*Bam*HI/or *Bam*HI/*Xba*I, and ligation into a *Sac*I/*Xba*I-digested pGP704, resulting in pGP*rfbU:*KanII. The correct chromosomal deletion of genes *rfbA-U* was confirmed by PCR.

Construction of Δ*epsC-N* mutant was done as previously described by Sikora et al. (2007) using the appropriate oligonucleotide pairs listed in the Table 2.

All expression plasmids were constructed in a similar manner. PCR fragments of the respective genes containing their own ribosomal binding sites were generated using the oligonucleotide pairs *x*_*y*_*z*_fwd and *x*_*y*_*z*_rev, where *x* represents the gene, *y* the restriction site/enzyme used and *z* the respective expression plasmid (Table 2). PCR fragments digested with the respective restriction enzymes were ligated into the similarly digested IPTG-inducible expression vectors pMMB67EH, pMMBneo or pQE60. Expression constructs were transformed into DH5αλpir, and Ap^r^ colonies were characterized by colony PCR.

### Immunoprecipitation

Immunoprecipitation was performed using Dynabeads^®^ Protein G Immunoprecipitation Kit (Invitrogen) according to manufacturer’s manual. 4 μl of npg1 antibody was incubated with 196 μl of binding and washing buffer and subsequently bound to the Dynabeads. Periplasmic protein fractions of respective strains were precipitated using ammonium sulfate and 200 μg of protein in 500 μl sample buffer was used as antigen. Proteins in the immunoprecipitations were incubated in 5× Laemmli buffer for 10 min at 100°C, separated with SDS-PAGE and analyzed by mass spectrometry.

### SDS-PAGE and Immunoblot Analysis

To separate proteins the standard sodium dodecyl sulfate-polyacrylamide gel electrophoresis (SDS-PAGE) procedure was used with 12 or 15% gels and the Prestained Protein Marker Broad Range (New England Biolabs) as a molecular mass standard. Proteins were visualized by conventional colloidal Coomassie staining according to Kang et al. (2002) or transferred to a nitrocellulose membrane (Amersham) for immunoblot analysis using mouse anti-His antisera (Qiagen, 1:2,000 diluted in 3% BSA) for detection of His-tagged proteins, anti-CT antisera (Sigma-Aldrich, 1:5,000 diluted in 10% skim milk), npg1 (1:10,000 diluted in 10% skim milk) or rabbit anti-BSA antisera (Thermo Fisher Scientific, 1:2,000 diluted in 3.7% soy milk) as primary antibodies. Peroxidase-conjugated goat anti-mouse (Dianova GmbH, diluted 1:7,500 in 10% skim milk) or peroxidase conjugated goat anti-rabbit (Dianova GmbH, 1:7,500 in 10% skim milk or 1:7,500 diluted in 3.7% soy milk in case of BSA detection) were used as secondary antibodies. Loading of equal amounts proteins was achieved by Bradford quantification and verified by Kang staining of SDS-PAGE gels in parallel to the immunoblot analysis. Chemiluminescence detection was performed by incubating the membrane in an ECL solution (Bio-Rad Laboratories) with subsequent exposure in a ChemiDoc XRS system (Bio-Rad Laboratories) in combination with the Quantity One software (Bio-Rad Laboratories).

### His-Purification of Proteins Using Immobilized Metal Ion Affinity Chromatography

Proteins with C-terminal His-tag were purified using a Ni Sepharose^TM^ Fast Flow (GE Healthcare) according to the manufacturer’s manual for batch purification. Briefly, appropriate amounts of *V. cholerae* cultures grown over night (ON) were inoculated in fresh LB to an OD_600_ of 0.1, grown to and OD_600_ of 0.5 following induction with IPTG (1 mM) for 6 h at 37°C. Cells were pelleted for 30 min at 17,000 × *g* and pellets were stored at −20°C. On the following day, pellets were thawed in 5 ml lysis buffer (150 mM NaCl, 50 mM Tris–HCl pH 7.5, 1% Triton-X) with the addition of protease inhibitor cocktail tablet (cOmplete^TM^, Roche). After sonification to lyse the cells, the soluble fraction was collected by centrifugation (30 min, 17,000 × *g*) and filtered through a syringe filter (0.45 μm). The resin was washed once with ddH_2_O and twice with binding and washing buffer pH 7.4 (81 mM Na_2_HPO_4_, 19 mM NaH_2_PO_4_, 0.5 M NaCl, 30 mM imidazole). Finally, 1 ml 50% Ni Sepharose slurry was added to 5 ml sample and incubated 1 h at 4°C with rotation. To wash the unbound protein, Ni Sepharose/sample was incubated three times 2 min with 1 mL binding and washing buffer on a rocker. Each time, Ni Sepharose/sample was collected by centrifugation 5 min at 500 × *g*. His-tagged protein was eluted stepwise with 500 μL elution buffer containing 100 mM, 200, 300or 500 mM imidazole, respectively. Each elution step was performed for 30 min at 4°C with rotation. All fractions were collected in the separate tubes and subjected to SDS-PAGE and/or immunoblot analyses.

### Protein Analysis by Mass Spectrometry

Protein bands excised from the SDS gel were reduced, alkylated and digested with Promega modified trypsin according to the method of Shevchenko et al. (1996). Peptide extracts were dissolved in 0.1% formic acid (FA), 5% acetonitrile (ACN) and separated by nano-HPLC (Dionex Ultimate 3000) equipped with an enrichment column (C18, 5 μm, 100 Å, 5 × 0.3 mm) and an Acclaim PepMap RSLC nanocolumn (C18, 2 μm, 100 Å, 500 × 0.075 mm) (all Thermo Fisher Scientific, Vienna, Austria). Samples were concentrated on the enrichment column for 6 min at a flow rate of 5 μl/min with 0.1% formic acid as isocratic solvent. Separation was carried out on the nanocolumn at a flow rate of 250 nl/min at 60°C using the following gradient, where solvent A is 0.1% formic acid in water and solvent B is acetonitrile containing 0.1% formic acid: 0–6 min: 4% B; 6–94 min: 4–25% B; 94–99 min: 25–95% B, 99–109 min: 95% B; 109.1–124 min: 4% B; The sample was ionized in the nanospray source equipped with stainless steel emitters (ES528, Thermo Fisher Scientific, Vienna, Austria) and analyzed in an Orbitrap velos pro mass spectrometer (Thermo Fisher Scientific, Waltham, MA, United States) operated in positive ion mode, applying alternating full scan MS (m/z 400–2,000) in the ion cyclotron and MS/MS by CID of the 20 most intense peaks with dynamic exclusion enabled. The LC-MS/MS data were analyzed by searching a database containing all *V. cholerae* sequences of the Uniprot database (downloaded on December 5th, 2016, 92555 sequences) and all common contaminants with Proteome Discoverer 1.4 (Thermo Fisher Scientific) and Mascot 2.4.1 (MatrixScience, London, United Kingdom). Carbamidomethylation on cysteine was entered as fixed and oxidation on methionine and 2,4-diacetamido-2,4,6-trideoxyhexose (DATDH) with a mass of 228.111 Da on serine as variable modification. Detailed search criteria were used as follows: semitrypsin; max. missed cleavage sites: 2; search mode: MS/MS ion search with decoy database search included; precursor mass tolerance ±10 ppm; product mass tolerance ±0.7 Da; acceptance parameters: 1% false discovery rate (FDR); only rank 1 peptides; minimum Mascot ion score 20; minimum 2 peptides per protein.

### PTM Analysis by Mass Spectrometry

Kang stained protein bands of affinity purified DegP was washed, de-stained and digested with both trypsin and GluC as previously described (Anonsen et al., 2012). Dried samples were redissolved in 0.1% FA prior to LC-MS/MS analysis. The LC-MS/MS analysis was performed as for protein analysis by MS with the following modification: Solvent B was changed to 90% ACN/9.9% H_2_O containing 0.1% FA. The column temperature was set at 40°C. The separation gradient was modified to, 0–3 min: 3% B; 3–53 min: 3–55% B; 55–59 min: 80% B, 60.3–63.3 min: 3% B. The sample was ionized in the nanospray source and analyzed in an Q Exactive Orbitrap mass spectrometer (Thermo Fisher Scientific, Waltham, MA, United States). Mass spectra were acquired in the positive ion mode applying alternating full scan MS (m/z 200–2,000) at a resolution of 70000 (at m/z 400) and MS/MS by HCD with NCE@28 of the 12 most intense peaks at a resolution of 17500 (at m/z 400) with dynamic exclusion for 30 s. Generated mass spectra were analyzed with the DegP protein sequence. Glycopeptides mass spectra were extracted based on the presence of the three diNAcBac reporter ions at m/z 229.118, at m/z 211.107 and at m/z 169.096 using Thermo Xcalibur (version 3.0.63) and manually assigned to DegP peptide sequences.

### Alkaline Phosphatase Assays

To determine the enzymatic activities for the transcriptional *phoA* fusions, alkaline phosphatase assays were performed as described previously (Manoil, 1991). Bacterial cultures were grown to mid-log phase (∼OD_600_ = 0.5) in LB media at 24°C. The activities were expressed in Miller units, given by (A_405_ × 1,000)/(A_600_ × ml × min).

### Static Biofilm Assay With Crystal Violet Staining

Biofilm assays under static conditions were essentially performed as previously described (Seper et al., 2011). Briefly, the respective strains were grown ON) in LB-Sm or LB-Ap/Glc liquid media (for plasmid containing strains). On the following day, a fresh culture LB-Sm or LB-Ap/IPTG (in case of strains harboring pMMB67EH or its derivatives), was adjusted to an OD_600_ = 0.01 and inoculated in a 96 well microtiter plate (U bottom, Sterilin). Biofilm was grown in a 24°C climate chamber for 24, 48 or 72 h. Wells were subsequently rinsed using a microplate washer (anthos Mikrosysteme GmbH, Fluido2). Biofilm was stained with 0.1% crystal violet, solubilized in 96% ethanol and the OD_595_ was measured (microplate reader: BMG Labtech SPECTROstar^Nano^) to quantify the amount of biofilm.

### Flow Cell Biofilm Experiments

For visualization and quantification of dynamically formed biofilm, a three channel flow cell system using 2% LB-Sm broth (24 h, RT) was used as described previously (Seper et al., 2011, 2014; Pressler et al., 2016). The respective GFP expressing *V. cholerae* strains were used for biofilm formation to allow acquisition of fluorescent images with confocal laser scanning microscopy. A coverslip 24 × 50 mm (Menzel-Glaeser) was used as substratum for biofilm growth. The respective ON cultures were adjusted to OD_600_ = 0.1 using 2% LB Approximately 300 μl of the dilutions were inoculated per channel. After static incubation for 2 h at RT, initial attachment of cells to the abiotic surface was analyzed in the attachment assay. For analysis of mature biofilm, the static incubation for 2 h at RT was followed by a constant flow of 2% LB at the rate of 3 ml h^–1^ for 22 h. Images of attached bacteria or biofilms were recorded by confocal laser scanning microscopy.

### Confocal Laser Scanning Microscopy

Images of biofilms were acquired using a Leica SP5 confocal microscope (Leica Microsystems, Mannheim, Germany) with spectral detection and a Leica HCX PL APO CS 63x water objective (NA 1.2). Optical sectioning was performed in 0.13 μm steps. GFP was excited at 488 nm, fluorescence emission was detected between 500 and 560 nm, and images were recorded without differential interference contrast (DIC) optics. For visualization and processing of image data the Leica LAS AF Lite and ImageJ 1.46 software was used. Quantification and morphological analysis of image stacks was performed using the computer program COMSTAT^1^ (Heydorn et al., 2000; Vorregaard, 2008).

### Cell Fractionation

A cell fractionation was performed essentially as described by Neu and Heppel (1965). Briefly, *V. cholerae* cultures grown ON were inoculated in fresh LB to an OD_600_ of 0.1, grown until OD_600_ of 0.5 and subsequently induced with 1 mM IPTG for 4 h. Supernatant (SUP) of the culture was collected by centrifugation 10 min, 5,000 × *g* and filter-sterilized using a 0.22 μm syringe filter. In parallel, cell equivalents reflecting 1 l of a culture with OD_600_ of 1 were pelleted by centrifugation (10 min, 5,000 × *g*) and washed in buffer 20 mM Tris–HCl pH 8. After another round of centrifugation, cells were resuspended in 20% sucrose and 1 mM Na-EDTA and incubated for 10 min. Cells were then centrifuged for 10 min, 5,000 × *g* at 4°C and resuspended in ice cold 0.5 mM Mg_2_SO_4_ by slow pipetting. The combination of cold temperature and hypotonic solution caused the burst of the bacterial outer membrane and release of the periplasmic fraction (PF) in the supernatant. After incubation for ∼5 min, samples were centrifuged for 5 min, 10,000 × *g* at 4°C to remove cell debris and intact cells. The supernatant was recovered as the periplasmic fraction. Periplasmic fractions were directly used for CT quantification by ELISA, while in all other cases proteins in the collected SUP and PF were either precipitated using trichloroacetic acid (TCA)/acetone for immunoblot analyses or ammonium sulfate in case of immunoprecipitation. In case of ELISA and immunoblot analyses, periplasmic fractions were controlled for cytoplasmic contamination and cell lysis via dot blot analyses detecting RpoA, the α-subunit of the RNA-Polymerase (for details see section “Dot Blot Analysis”). A representative dot blot is shown in Supplementary Figure S4A. In general, similar intensities for periplasmic fractions used for ELISA were observed. Moreover, intensities in all periplasmic fractions were magnitudes lower compared to whole cell extracts (WCE). This indicates equally low levels of cytoplasmic contamination for all periplasmic fractions used for ELISA.

### Dot Blot Analysis

Dot blot analyses was essentially performed as described recently using a 96-well Whatman Minifold I Dot-Blot System (GE Healthcare) (Roier et al., 2016). Briefly, equal protein amounts (according to Bradford) of periplasmic factions or dilutions (3-fold steps) thereof were spotted onto a nitrocellulose membrane. Dilutions (3-fold steps) of a whole cell extract derived from the WT served as positive control (for details see section “Generation of Whole Cell Extracts”). Then, each membrane was dried, incubated in TBS (20 mM Tris/HCl, pH 7.5, 150 mM NaCl) for 2 min, and blocked in 10% skim milk in TBS for 2 h. Afterward, the membrane was incubated with a monoclonal antibody to the α-subunit of the RNA polymerase (RpoA, NeoClone Biotechnology, diluted 1:2,000 in 10% skim milk) overnight. The next day, membranes were washed three times in TBS for 10 min, incubated with the secondary antibody (horseradish peroxidase-conjugated goat anti-mouse IgG antibody from Dianova, diluted 1:7,500 in 10% skim milk in TBS) for 2 h, washed once in TBS-T (20 mM Tris/HCl, pH 7.5, 250 mM NaCl, 0.05% Tween-20), and twice in TBS for 10 min each. Finally, chemiluminescence detection was performed as described above (for details see section “SDS-PAGE and Immunoblot Analysis”).

### Precipitation of Proteins Using TCA/Acetone

Protein precipitation using TCA and acetone was essentially performed as described by Link and LaBaer (2011), with following modifications. Samples were spiked with BSA (0.1 μg/ml), before a solution of 100% TCA was added to the protein samples (SUP or the PF) to the final TCA concentration of 20%. Samples were mixed and stored ON at −20°C. On the next day, samples were thawed and centrifuged 30,000 × *g* for 1 h. Supernatant was carefully decanted and 20 ml of acetone was added to the pellet. Samples were again centrifuged 30,000 × *g* for 1 h. A second washing step with 20 ml of acetone was performed with the final centrifugation step of 50,000 × *g*. After decanting the supernatant, pellets were air-dried for 30 min and subsequently suspended in 200 μl of PBS buffer. In attempt to completely dissolve the pellet, samples were placed in a sonification bath for 30 min. Undissolved particles were removed by short centrifugation. Concentration of proteins was determined with Bradford assay (BioRad). Similar efficiency of protein precipitation using TCA was controlled by immunoblot analyses detecting BSA (for details see section “SDS-PAGE and Immunoblot Analysis”).

### Precipitation of Proteins Using Ammonium Sulfate

Periplasmic protein content was also subjected to precipitation using ammonium sulfate, which yields a relatively soluble protein extract essential for immunoprecipitation. Following cell fractionation, ammonium sulfate was added to the periplasmic fraction to final concentration of 50% (Wood, 1976) and stirred for 1 h at RT. Sample was then subjected to centrifugation 10,000 × *g* for 30 min at RT. After decanting supernatant, pellet was suspended in 500 μl PBS buffer. Sample was dialyzed ON against PBS in a dialysis tube with pore size 3.5 (Roth).

### Growth Assays

Growth kinetics were performed as previously described in M9-chitin or M9-GlcNAc with following modifications (Schild et al., 2007). Briefly, Δ*pglL*_Vc_Δ*rbmD* or WT were grown in a M9-Glc pre-culture ON and inoculated in M9-chitin or M9-GlcNAc to a starting OD_600_ = 0.025. To determine the exact CFUs appropriate dilutions were plated on LB plates at 0, 24, 48, and 72 h. Before plating each culture was rigorously mixed by vortexing. The cultures were incubated at 37°C with aeration and samples were collected after 24, 48, and 72 h. Results are given as CFU/ml normalized to a starting concentration of 10^7^ CFU/ml at *t* = 0 h.

### Generation of Whole Cell Extracts

Whole cell extracts were generated by collecting the pellet of 20 ml culture by centrifugation 5 min at 5,000 × *g*. Pellet was suspended in 1 ml of PBS buffer and transferred to a cryotube. Glass beads (0.5 mm in diameter, Roth) were added to the sample and lysed using the PowerLyser^TM^ Bench Top Bead-Based Homogenizer (MO BIO Laboratories, Inc.) 3 times 1 min, with 1 min incubation steps on ice after each round of lysis. Samples were centrifuged 2 min at 10,000 × *g* to remove cell debris and unbroken cells. Supernatant was separated from glass beads and used as WCE.

### CT ELISA

Cholera toxin production in culture supernatants or periplasmic fractions was determined by the ganglioside G_M__1_ ELISA (Pressler et al., 2016). *V. cholerae* strains were grown ON in LB medium supplemented with 5% glucose, which is beneficial for growth of the Δ*epsC-N* (Sikora et al., 2007). On the following day, strains were shifted to AKI broth with 5% glucose, which represents virulence gene inducing conditions. Strains were grown at 37°C for 4 h anaerobically, followed by 4 h aerobic growth with shaking at 180 rpm (Iwanaga et al., 1986). For complementation strains, which have been grown and induced with IPTG on 37°C, neither LB medium nor AKI broth contained additional glucose to avoid interference with P_*tac*_ promotor expression. Subsequently, cells were removed from CT containing supernatants by centrifugation and supernatants were stored at –20°C. Periplasmic fractions were obtained from the cell pellet as described above (see section “Cell Fractionation”) and adjusted to protein equivalents (Bradford). ELISA plates (BRANDplates^®^, 96-well, immunoGrade^TM^) were coated with 10 μg/ml G_M__1_ ganglioside (Calbiochem) in 60 mM Na_2_CO_3_ ON at 37°C and washed four times with PBS pH 7.4 and 0.5% Tween (PBS-T). Free binding sites were blocked with 4 mg/ml BSA in PBS (BSA-PBS) for 1 h at RT. After washing as described above, CT containing supernatants were diluted in PBS and added to the plate. Additionally, purified CT in PBS (Sigma-Aldrich) was inoculated in separate wells to generate a standard curve. ELISA plates were incubated with supernatants and purified CT for 1 h at RT and were washed again as described above. After incubation with the primary antibody (Sigma-Aldrich, anti-CT antibody), diluted 1:10,000 in BSA-PBS for 1 h at RT, ELISA plates were washed four times with PBS-T. After incubation with the secondary antibody (Dianova GmbH, peroxidase conjugated goat anti-rabbit), diluted 1:2,000 in BSA-PBS for 1 h at RT and subsequent washing, the ELISA plates were incubated with TMB Substrate Reagent Set (BioLegend, Vienna) for detection of CT. The reaction was stopped by the addition of 1 M H_3_PO_4_ and ELISA plates were measured at OD_450_ by using SPECTROstar^Nano^ microplate reader (BMG Labtech).

### Statistical Analysis

Data were analyzed using the Mann–Whitney *U* test or a Kruskal–Wallis test followed by *post hoc* Dunn’s multiple comparisons. Differences were considered significant at *P*-values of <0.05. For all statistical analyses, GraphPad Prism version 6 was used.

## Results

### Reverse Glycoengineering Reveals That PglL_Vc_ Glycosylates Multiple *V. cholerae* Proteins

**FIGURE 1:**
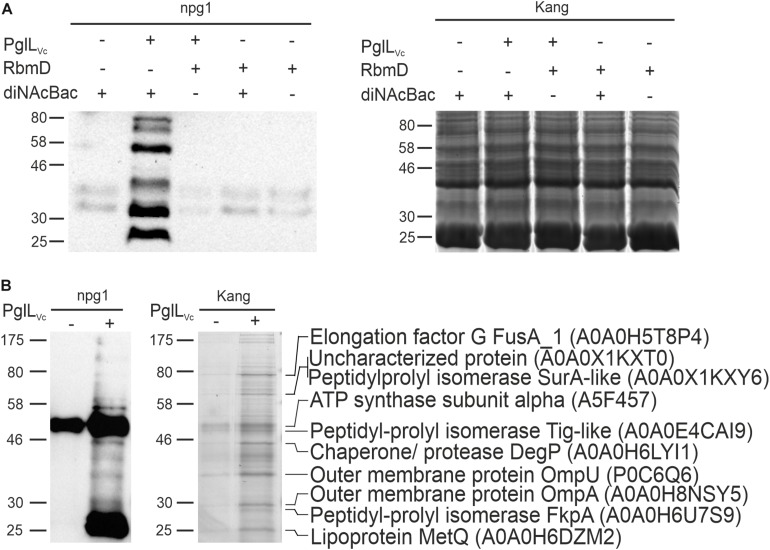
Expression of the diNAcBac glycan in *V. cholerae* results in multiple glycosylated proteins in a PglL_Vc_-dependent manner. **(A)** Shown is a representative immunoblot using the anti-diNAcBac antibody (npg1) to detect diNAcBac-glycosylated proteins and the corresponding Kang-stained SDS-gel of whole cell extracts derived from Δ*pglL*_Vc_Δ*rbmD* with or without *in trans* expression of respective *O*-OTases PglL_Vc_ or RbmD (+ = pMMBneo-pglL_Vc_ or pMMBneo-rbmD; – = pMMBneo) as well as the diNAcBac glycan (+ = pACYCpglFBCD; – = pACYC184). Only the presence of PglL_Vc_ and diNAcBAc yielded in the detection of multiple bands. **(B)** Shown is an immunoblot using the npg1-antibody to detect diNAcBac-glycosylated proteins and the corresponding Kang-stained SDS-gel of immunopreciptated samples of Δ*pglL*_Vc_Δ*rbmD* expressing the diNAcBac glycan (pACYCpglFBCD) in the presence (+ = pMMBneo-pglL_Vc_) or absence (- = pMMBneo) of PglL_Vc_. Immunoprecipitation was performed using npg1 immobilized onto Dynabeads coupled with protein G in combination with membrane and periplasmic enriched protein samples from the respective strains. Selected protein bands, which appear to be enriched upon presence of PglL_Vc_ on the SDS-gel and resulted in a decent signal on the immunoblot, were excised and subjected to MS. Identified proteins are indicated on the right with their respective position on the gel, protein identities and accession numbers. **(A,B)** Lines to the left indicate the molecular masses of the protein standards in kDa.

The activity of PglL_Vc_ has only been shown in a heterologous *E. coli* system via simultaneous expression of PglL_Vc_ along with a defined glycan and protein acceptors for *O*-linked protein glycosylation from foreign bacteria (Gebhart et al., 2012). Although this demonstrated functionality of PglL_Vc_ with relaxed glycan specificity, *O*-linked protein glycosylation has not been documented in *V. cholerae*. Hence, neither the transferred glycan structure nor glycosylated proteins of *V. cholerae* are known. Along this study we focused on the latter and exploited the relaxed glycan specificity of PglL_Vc_ along with a well-characterized glycan biosynthesis locus from *N. gonorrhoeae* to identify a first set of glycosylated proteins in *V. cholerae.* Therefore, we introduced a plasmid harboring the core *pglFBCD* glycosylation locus from *N. gonorrhoeae* into *V. cholerae* to express the lipid-linked monosaccharide precursor Und-PP-diNAcBac. Proteins glycosylated with diNAcBac monosaccharide could then be readily detected using the specific monoclonal antibody npg1 (Aas et al., 2007; Faridmoayer et al., 2007; Egge-Jacobsen et al., 2011; Anonsen et al., 2015). Immunoblotting of WCE revealed a number of distinct npg1-reactive proteins whose detection was dependent on the presence of both PglL_Vc_ and the *N. gonorrhoeae pgl* plasmid (Figure 1A). Moreover, these findings were independent on the presence or absence of RbmD. This indicates that PglL_Vc_, in contrast to RbmD, can utilize the diNAcBac glycan to modify a defined subset of *V. cholerae* proteins. Hence, we focused on the identification of proteins glycosylated via PglL_Vc_.

To identify *V. cholerae* proteins bearing the diNAcBac monosaccharide, immunoaffinity purification was employed as previously described (Anonsen et al., 2012) using samples enriched for membrane and periplasmic proteins from various backgrounds as prey (Figure 1B). Immunoblotting using the npg1 monoclonal antibody of immunoprecipitated samples revealed a number of proteins specifically detected in the presence of both PglL_Vc_ and the *N. gonorrhoeae pgl* plasmid. Besides several bands with low intensity the immunoblot shows two intense bands at approximately 50 and 25 kDa. The 50 kDa band is likely a cross-reacting band as it also detected in absence of PglL_Vc_. In contrast, the 25 kDa band only appears in presence of PglL_Vc_, but its exact nature remains to be elucidated.

Robust signals obtained by immunoblot indicating immunoaffinity purified proteins or complexes thereof, were aligned to protein bands on a conventionally stained gel that were subsequently excised and identified by mass spectrometric (MS) techniques. The analyses revealed several proteins, such as abundant outer membrane proteins OmpA and OmpU as well as periplasmic chaperones, i.e., the peptidyl-prolyl *cis*-*trans* isomerases (SurA-like and FkpA) and the chaperone/protease DegP (Figure 1B).

### DegP Glycosylation Can Be Reconstituted in an *E. coli* Background

**FIGURE 2:**
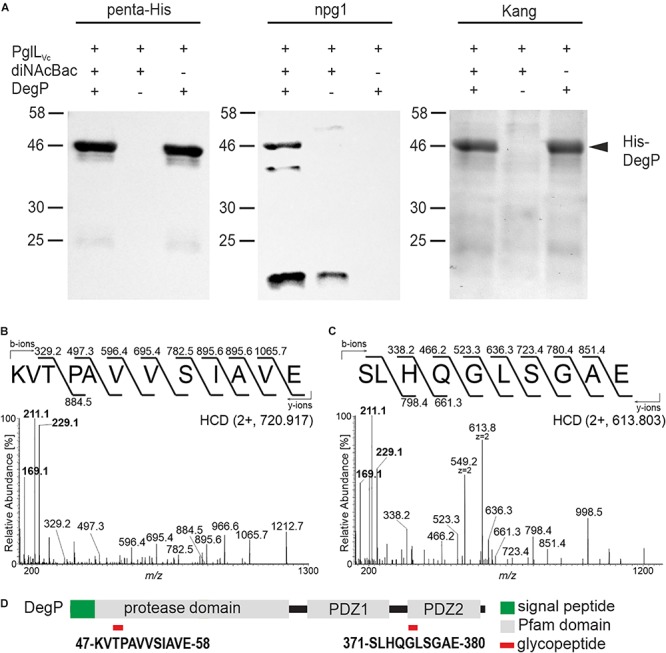
PglL_Vc_ glycosylates DegP with the diNAcBac glycan. **(A)** Ni-sepharose purified samples of *E. coli* CLM24 with varying expression of PglL_Vc_ (pMMBneo-pglL_Vc_), the diNAcBac glycan (+ = pACYCpglFBCD; – = pACYC184) and/or His-tagged DegP (+ = pQE60-degP; – = pQE60), respectively, were subjected for immunoblot analyses using the penta-His antibody or the npg1 antibody. A corresponding Kang-stained SDS-gel served as control. The arrow highlights the location of the His-DegP band (approx. 46 kDa). Only in the presence of PglL_Vc_, diNAcBac, and His-DegP a band of similar size can be detected with the npg1 antibody, suggesting a diNAcBac-modification of His-DegP. Lines to the left indicate the molecular masses of the protein standards in kDa. **(B,C)** Identification of GluC-derived glycopeptides from DegP. HCD MS/MS spectra of the [M+2H+]^2+^ precursor ions at m/z 720.917 and 613.803 of the ^47^KVTPAVVSIAVE^58^ and ^371^SLHQGLSGAE^380^ peptides respectively, modified with one glycan. Spectra show the reporter ions of diNAcBac at m/z 229.1, at m/z 211.1 and at m/z 169.1 in the low mass area. **(D)** Domain organization and location of DegP glycopeptides. Glycopeptides are numbered according to the DegP sequence (generated by in-house sequencing). Red rectangles denote the location of the glycopeptide.

In order to further validate the glycosylation status of the proteins identified above, the ability of DegP as a representative candidate to be glycosylated in a heterologous system was examined. Plasmids individually expressing PglL_Vc_, a C-terminal His-tagged DegP and Und-PP-diNAcBac were introduced into the glycosylation-deficient *E. coli* strain CLM24. Following a His-tag purification, samples were subjected to immunoblot analyses using penta-His or npg1 antibodies as well as conventional gel staining (Figure 2A). Co-expression of PglL_Vc_ and Und-PP-diNAcBac was both necessary and sufficient to establish reactivity of DegP with npg1, indicative of diNAcBac modification (Figure 2A). In addition, a cross-reactive band (∼20 kDa) was detected by the npg1 antibody upon expression of the diNAcBac. It could be speculated that this band reflects the lipid-linked monosaccharide precursor Und-PP-diNAcBac (Und-PP-diNAcBac), but the exact origin remains to be elucidated.

Gel slices bearing His-tagged DegP were excised and processed for MS analyses to detect glycan attachment sites. Utilizing LC-MS/MS employing higher-energy collision dissociation in conjunction with in-gel digestion with trypsin and GluC (HCD) (Anonsen et al., 2012) peptides covering 98% of DegP including two glycopeptides were identified (Supplementary Figure S2A). Specifically, the MS/MS spectrum of the doubly charged precursor ion at *m*/*z* 720.917 (charge adjusted mass 1440.827 [M+H+]) corresponds to the peptide ^47^KVTPAVVSIAVE^58^ (theoretical monoisotopic mass 1212.720 [M+H+]) modified with a single diNAcBac moiety (228.111 Da) (Figure 2B). Similarly, the MS/MS spectrum of the doubly charged precursor ion at *m*/*z* 613.803 (charge adjusted mass 1226.599 [M+H+]) corresponds to the peptide ^371^SLHQGLSGAE^380^ (theoretical monoisotopic mass 998.490 [M+H+]) with a single diNAcBac modification (Figure 2C). In addition to extensive *y*- and *b*-ions series that were detected in both MS/MS spectra enabling the accurate amino acid sequence determination for both peptides, the diNAcBac specific reporter ions at m/z 229.118, at m/z 211.107 and at m/z 169.096 (Anonsen et al., 2012) was detected in the low mass area. The MS results therefore clearly establish both the ^47^KVTPAVVSIAVE^58^ and the ^371^SLHQGLSGAE^380^ peptides as modified by a diNAcBac moiety at a single site (Figures 2B,C and Supplementary Figures S2B,C).

Interestingly, both DegP glycopeptides are located within defined domain structures. The ^47^KVTPAVVSIAVE^58^ glycopeptide is localized within the protease domain whereas the ^371^SLHQGLSGAE^380^ glycopeptide is positioned in the carbohydrate binding loop of the PDZ2 domain (Figure 2D), which is suggested to be involved in substrate binding (Krojer et al., 2002). Thus, if identical sites of glycan attachment occur in *V. cholerae*, DegP activities might be altered.

### PglL_Vc_ and RbmD Status Affect Secretion of Cholera Toxin

Based on the confirmed *O*-glycosylation of DegP, the identification of other periplasmic chaperones to be targets for *O*-glycosylation in *V. cholerae* (Figure 1B) and a recent report implying that DegP affects type II-dependent secretion in *V. cholerae* (Altindis et al., 2014), we sought to examine if PglL_Vc_ influences T2SS-associated phenotypes. Given the structural similarities of RbmD to PglL_Vc_ and other established oligosaccharyltransferases (Supplementary Figure S1) we also examined the potential effects of RbmD, although the *O*-OTase activity of RbmD remains to be confirmed. Thus, respective single and double mutants (i.e., Δ*pglL*_Vc_, Δ*rbmD*, and Δ*pglL*_Vc_Δ*rbmD*) as well as complementation strains for both gene products were included in the following phenotypical analyses.

**FIGURE 3:**
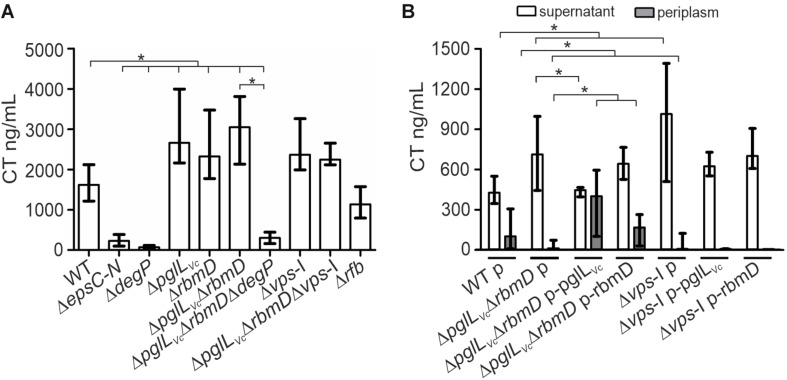
*O*-OTases impact CT secretion. **(A)** The amount of secreted CT was determined in the culture supernatants by ELISA for *V. cholerae* WT, Δ*epsC-N*, *ΔdegP*, Δ*pglL*_Vc_, Δ*rbmD*, Δ*pglL*_Vc_Δ*rbmD*, Δ*pglL*_Vc_Δ*rbmDΔdegP*, Δ*vps*-I, Δ*pglL*_Vc_Δ*rbmD*Δ*vps*-I, and Δ*rfb.* Strains were grown under virulence gene factor expressing conditions (AKI conditions) to induce CT expression. **(B)** The amounts of secreted (open bars) and periplasmic (gray bars) CT for strains carrying empty vector or complementation plasmids were determined in the culture supernatants and periplasmic fractions by ELISA. Periplasmic fractions were controlled for cytoplasmic contamination and cell lysis via dot blot analyses detecting RpoA, the α-subunit of the RNA-Polymerase (for details see section “Dot Blot Analysis”). A representative dot blot is shown in Supplementary Figure S4A, indicating equally low levels of cytoplasmic contamination for all periplasmic fractions. **(A,B)** The data is given as median with interquartile range (*n* ≥ 8). Asterisks highlight significant differences between respective data sets (^∗^*P* < 0.05 Kruskal–Wallis test followed by *post hoc* Dunn’s multiple comparisons).

*V. cholerae* secretes several important T2SS effectors along its lifecycle (Sikora, 2013). The most prominent example is probably CT, which is predominantly responsible for the secretory diarrhea during human colonization. To investigate whether secretion of CT is affected in the context of the PglL_Vc_, and RmbD status, secretion levels of CT were compared for WT, *ΔdegP*, Δ*pglL*_Vc_, Δ*rbmD*, Δ*pglL*_Vc_Δ*rbmD*, and Δ*pglL*_Vc_Δ*rbmDΔdegP*. As a control, CT secretion was measured in a Δ*epsC-N* background that lacks essential parts of the T2SS machinery and is therefore severely impaired for secretion. Of note, CT expression remained equal in all strains tested as indicated by comparable signal intensities in the WCE of respective strains (Supplementary Figure S3A). Quantification of secreted CT revealed significant higher levels in the supernatants of Δ*pglL*_Vc_, Δ*rbmD*, and Δ*pglL*_Vc_Δ*rbmD* backgrounds compared to WT (Figure 3A).

Restoration of PglL_Vc_ expression significantly lowered the CT secretion compared to the Δ*pglL*_Vc_Δ*rbmD* with an empty vector control, while with restoration of RbmD expression, only a mild reduction was observed (Figure 3B). In addition, the CT levels in the periplasm were quantified for theses strains showing an inverse pattern compared to secreted CT. Periplasmic CT levels were significantly lower for Δ*pglL*_Vc_Δ*rbmD* with an empty vector compared to WT with empty vector. Moreover, PglL_Vc_ or RbmD expression significantly increased periplasmic CT levels compared to Δ*pglL*_Vc_Δ*rbmD* with an empty vector. Again, equal CT expression was observed in these strains with plasmids indicated by comparable signal intensities in the respective WCE (Supplementary Figure S3B). In comparison to the WT a marked decrease of CT secretion for the T2SS-mutant Δ*epsC-N* and *ΔdegP* was observed (Figure 3A). Moreover, deletion of *degP* in the Δ*pglL*_Vc_Δ*rbmD* background (Δ*pglL*_Vc_Δ*rbmDΔdegP*) also reduced CT secretion compared to Δ*pglL*_Vc_Δ*rbmD*. Thus, *degP* is epistatic to *pglL*_Vc_ and *rbmD*. In summary, CT is more efficiently secreted in the absence of PglL_Vc_ and RbmD, while presence of PglL_Vc_ and RbmD is associated with reduced translocation into the supernatant resulting in accumulation of CT in the periplasm.

Next, we tried to pinpoint the potential origin of an endogenous protein glycosylation by assuming that a defect in endogenous glycan availability might phenocopy *pglL_Vc_-* and *rbmD-*mutants. In principle, *V. cholerae* encodes two major glycan biosynthesis pathways, represented by the *vps-* (*Vibrio* exopolysaccharide, VPS) and *rfb-* (O-antigen glycan) gene clusters (Manning et al., 1995). To assess the potential influence of these two pathways, CT secretion was quantified for strains lacking either the *rfb* or *vps* genes. Similar to Δ*pglL*_Vc_, Δ*rbmD*, and Δ*pglL*_Vc_Δ*rbmD*, mutants lacking the entire *vps*-I gene cluster (Δ*vps-*I and Δ*pglL*_Vc_Δ*rbmD*Δ*vps-*I) showed increased CT levels in the supernatant compared to WT, whereas deletion of *rfb* genes (Δ*rfb*) had no effect (Figure 3A). Deletion of *pglL*_Vc_ and *rbmD* in addition to the *vps*-I gene cluster did not further enhance the secreted CT levels. Moreover, Δ*vps-*I and Δ*pglL*_Vc_Δ*rbmD* showed a similar secretion efficacy of CT with almost no detectable CT amounts in the periplasm and high levels in the supernatant (Figure 3B). Notably, expression of PglL_Vc_ and RbmD in Δ*vps-*I (Δ*vps-*I p-pglL_Vc_ and Δ*vps-*I p-rbmD) did not restore detectable CT levels in the periplasm (Figure 3B). These phenotypes provide a first hint that the *vps* cluster could be involved in providing an endogenous glycan.

### PglL_Vc_ and RbmD Affect Growth on Chitin

**FIGURE 4:**
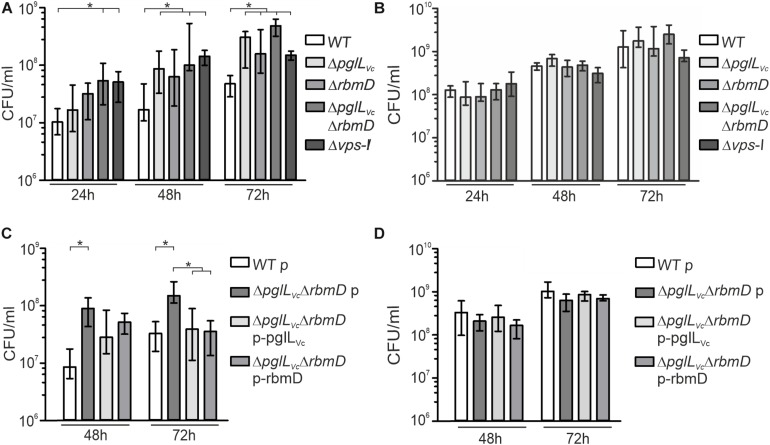
Deletion of *O*-OTases affects chitin utilization. **(A,B)** Shown is growth (CFU/ml) at indicated time points of WT, Δ*pglL*_Vc_, Δ*rbmD*, Δ*pglL*_Vc_Δ*rbmD*, and Δ*vps*-I in minimal media M9 supplemented with chitin **(A)** or supplemented with GlcNAc **(B)** as a sole carbon source. **(C,D)** Shown is growth (CFU/ml) at indicated time points of WT with empty vector (WT p), Δ*pglL*_Vc_Δ*rbmD* with empty vector (Δ*pglL*_Vc_Δ*rbmD* p), Δ*pglL*_Vc_Δ*rbmD* p-pglL_Vc_, and Δ*pglL*_Vc_Δ*rbmD* p-rbmD in minimal media M9 supplemented with chitin **(C)** or supplemented with GlcNAc **(D)** as a sole carbon source. **(A–D)** The data is given as median with interquartile range (*n* ≥ 8). Asterisks highlight significant differences between respective data sets (^∗^*P* < 0.05 Kruskal–Wallis test followed by *post hoc* Dunn’s multiple comparisons).

In the aquatic lifestyle, *V. cholerae* secrets several proteins of the chitin utilization program via the T2SS (Sikora et al., 2011). As PglL_Vc_ and RbmD may generally affect secretion of T2SS-dependent substrates including chitinases, mutants thereof might exhibit altered growth on chitin. Therefore, we compared growth dynamics of Δ*pglL*_Vc_, Δ*rbmD*, and Δ*pglL*_Vc_Δ*rbmD* to the WT in minimal media supplemented with chitin or its corresponding monomer GlcNAc as sole carbon sources, respectively (Figures 4A,B). All strains showed similar growth on GlcNAc demonstrating equal uptake and metabolic usage of the monosaccharide (Figure 4B). In contrast, the Δ*pglL*_Vc_, Δ*rbmD* and especially the Δ*pglL*_Vc_Δ*rbmD* mutant showed enhanced growth on chitin that gradually increased over the course of 72 h growth period compared to the WT (Figure 4A). Similar to Δ*pglL*_Vc_Δ*rbmD* mutant, the Δ*vps-*I mutant also showed enhanced growth on chitin, but similar growth on GlcNAc, if compared to the WT (Figures 4A,B). *In trans* expression of both PglL_Vc_ and RbmD in the double mutant did not affect growth on GlcNAc, but significantly reduced growth on chitin and allowed (partial) restoration of the WT phenotypes at 48 and 72 h (Figures 4C,D). In conclusion, single and double mutants of *pglL*_Vc_ and *rbmD* have a fitness advantage compared to WT while growing on chitin, consistent with the notion of a more efficient secretion of chitinases as T2SS substrates.

### PglL_Vc_ and RbmD Affect Biofilm Formation

**FIGURE 5:**
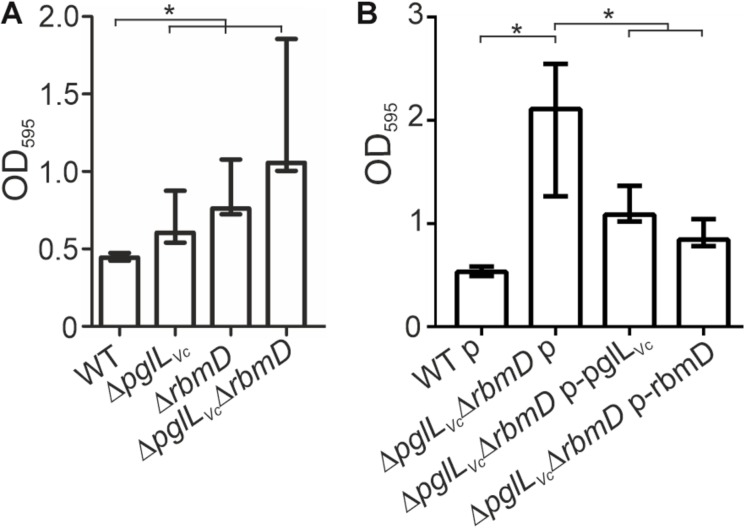
*O*-OTases impact static biofilm formation. The biofilm formation capacity of the strains indicated on the *x*-axis was assayed under static conditions by crystal violet staining and subsequent determination of the OD_595_ (*n* ≥ 24). **(A)** Biofilms of WT, Δ*pglL*_Vc_, Δ*rbmD* and Δ*pglL*_Vc_Δ*rbmD* were quantified after 48 h. **(B)** *In trans* expression of PglL_Vc_ or RbmD partially restores biofilm to WT levels at 48 h. **(A,B)** The data is given as median with interquartile range (*n* ≥ 24). Asterisks highlight significant differences between the indicated data sets (^∗^*P* < 0.05 Kruskal–Wallis test followed by *post hoc* Dunn’s multiple comparisons).

Both, *pglL*_Vc_ and *rbmD*, have been previously identified in a screen for genes up-regulated during dynamic biofilm formation (Seper et al., 2014). In that report, they were excluded from subsequent characterization as they did not meet the stringent criteria for bona fide in biofilm induced and were at time only annotated with unknown function. Of note, *rbmD* is part of the *rbm*-gene cluster (*r*ugosity and *b*iofilm structure *m*odulators) that harbors the biofilm matrix protein genes *rbmA* and *rbmC* (Fong et al., 2006; Fong and Yildiz, 2007). Based on this, we analyzed the *pglL*_Vc_ and *rbmD* mutants for alterations in biofilm formation. First, we focused on static biofilm formation after 24 and 48 h, representing two time points frequently used for biofilm analyses in *V. cholerae* (Seper et al., 2011). In general, altered biofilm formation can already be observed at 24 h (Supplementary Figure S5A), but phenotypes are more pronounced after 48 h (Figure 5A). Here, the Δ*pglL*_Vc_ mutant showed a slight, but significant increase in static biofilm formation, while the Δ*rbmD* mutant and especially the Δ*pglL*_Vc_Δ*rbmD* double mutant exhibited pronounced increases in biofilm formation compared to the WT (Figure 5A). *In trans* expression of both PglL_Vc_ and RbmD in the double mutant significantly reduced biofilm formation and allowed partial restoration of the WT phenotypes at both time points (Figure 5B and Supplementary Figure S5B).

**FIGURE 6:**
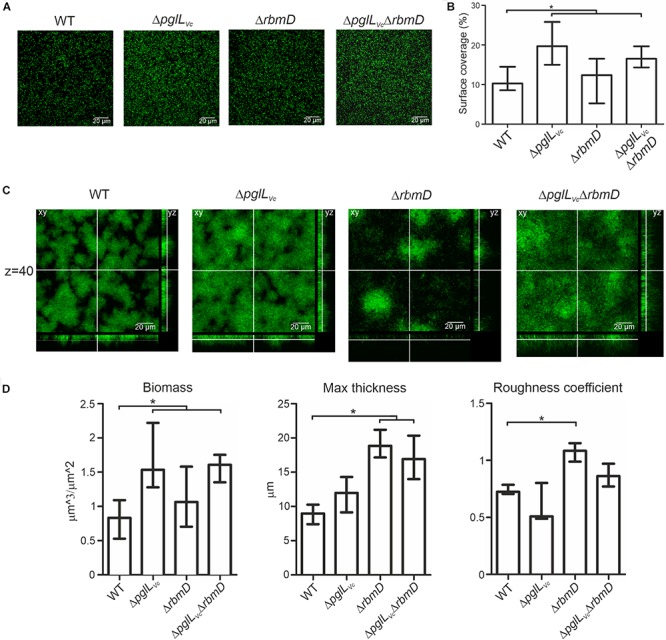
*O*-OTases alter attachment on abiotic surfaces and 3-dimensional biofilm architecture. **(A)** Shown are representative confocal microscopy images of surface coverage after 2 h incubation time reflecting attachment properties. **(B)** The median surface coverage of GFP-expressing WT and deletion mutants after 2 h incubation in flow cell chambers was determined by the COMSTAT software (*n* ≥ 6). **(C)** Shown are representative confocal laser scanning microscopy images of GFP-expressing WT and deletion mutant biofilms as horizontal (*xy*) and vertical (*xz* and *xy*) projections. Biofilms were allowed to mature for 24 h in flow cell chambers with constant 2% LB medium flow. The xy panels represent selected single optical sections through the three-dimensional data sets at the indicated *z* position (*z* = 40, steps = 0.13 μm). **(D)** Biofilm parameters such as biomass, the maximum thickness and the roughness coefficient of WT and mutants were analyzed using COMSTAT software (*n* ≥ 13). **(A,C)** Images were obtained with Leica SP5 confocal microscope. **(B,D)** Data is presented as median with interquartile range. Asterisks highlight significant differences between respective data sets (^∗^*P* < 0.05 Kruskal–Wallis test followed by *post hoc* Dunn’s multiple comparisons).

Three-dimensional biofilm morphology was microscopically analyzed in a flow cell system using GFP-expressing backgrounds (Seper et al., 2014). The Δ*pglL*_Vc_ mutant and the Δ*pglL*_Vc_,Δ*rbmD* double mutant exhibited a significant increased attachment capability, while the surface coverage of the Δ*rbmD* mutant was unaltered compared to the WT (Figures 6A,B). In mature biofilms, distinct differences could be observed upon deletion of *pglL*_Vc_ and *rbmD* (Figure 6C). Most pronounced phenotypes in comparison to the WT were the increased biomass in case of Δ*pglL*_Vc_ biofilms and the enhanced maximum thickness and higher roughness coefficient for Δ*rbmD* biofilms (Figure 6D). Biofilm formation of the double mutant Δ*pglL*_Vc_Δ*rbmD* combines characteristics of both single mutants with an increase in attachment, biomass and maximum thickness (Figure 6D). *V. cholerae* mutants exhibiting increased biofilm formation can be frequently correlated with enhanced expression of *vps* genes encoding proteins for the *V. cholerae* exopolysaccharide (VPS) matrix synthesis and secretion (Yildiz and Schoolnik, 1999). To assess whether the enhanced biofilm formation was related to enhanced *vps* expression here, chromosomal transcriptional fusions of a promoterless *phoA* reporter gene to *vpsA* representing one of the first genes in the *vps-*I locus, were constructed in the WT and mutant backgrounds, as well as in the Δ*hapR* mutant, which served as a positive control for de-repressed *vpsA* expression (Seper et al., 2011). Thus, PhoA activities reflect the transcription levels of *vpsA* in the respective strains. *O*-OTase mutants and WT exhibited comparable levels of PhoA activity indicating similar transcription levels of *vpsA* in these strains (Supplementary Figure S5C). Thus, the alterations in biofilms and attachment cannot be simply explained by elevated transcription of VPS genes.

### RbmD Restricts RbmA Secretion in a VPS Dependent Manner

**FIGURE 7:**
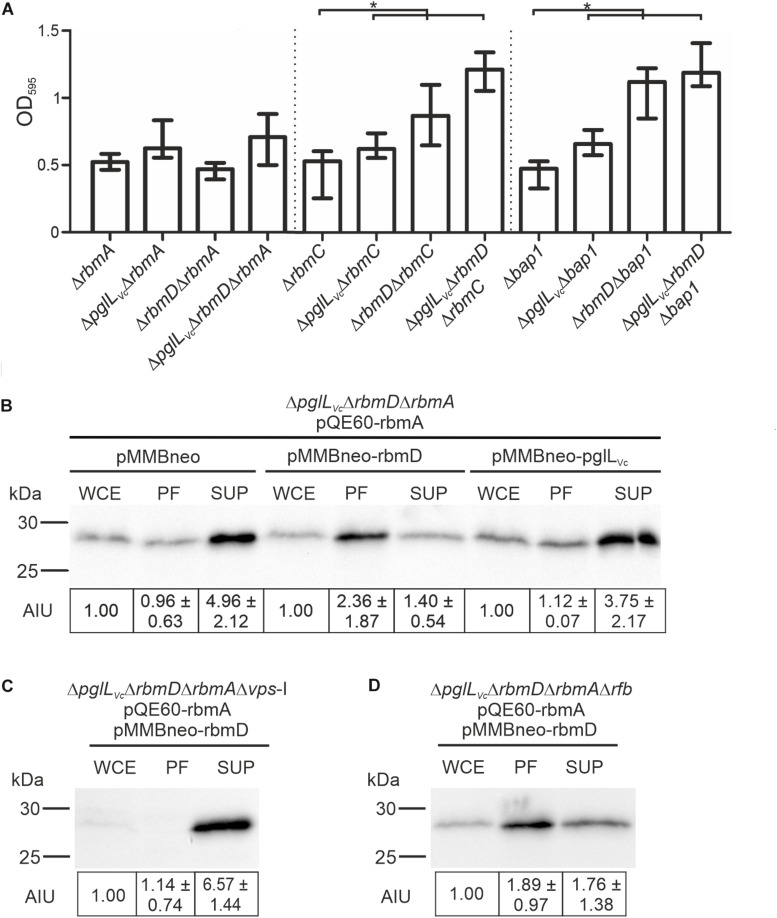
*O*-OTases impact RbmA secretion. **(A)** Deletion of *rbmA*, but not *rbmC* or *bap1*, negates the increased biofilm formation in *O*-OTase mutants. The biofilm formation capacity of the strains indicated on the *x*-axis were quantified after 48 h under static conditions by crystal violet staining and subsequent determination of the OD_595_ (*n* ≥ 24). **(B–D)** Shown are representative immunoblots detecting His-RbmA in whole cell extracts (WCE), periplasmic fractions (PF), and supernatants (SUP) of strain Δ*pglL*_Vc_Δ*rbmD*Δ*rbmA* **(B)**, Δ*pglL*_Vc_Δ*rbmD*Δ*rbmA*Δ*vps*-I **(C)**, or Δ*pglL*_Vc_Δ*rbmD*Δ*rbmA*Δ*rfb* **(D)** expressing His-tagged RbmA *in trans* (pQE60-rbmA) in the presence or absence of RbmD (pMMBneo-rbmD or pMMBneo), respectively. Equal amounts of proteins for WCE, PF or SUP were loaded according to Bradford to allow direct comparison of the fractions. Semiquantitative densitometric evaluation of detected His-RbmA was performed with the Quantity One software (Bio-Rad Laboratories) and is indicated below the immunoblots as arbitrary intensity units [mean AIU with standard deviation (*n* ≥ 3)] normalized to WCE of the respective strain, which was always set to 1. Notably, TCA precipitation of PF and SUP was necessary to ensure stable detection. Similar efficiency of protein precipitation for PF and SUP using TCA was controlled by spiking the samples with BSA prior to the precipitation and subsequent immunoblot analyses detecting BSA in the precipitated samples. A representative immunoblot is shown in Supplementary Figure S6, indicating equal precipitation efficiency in all samples. Moreover, PF obtained after TCA precipitation were controlled for differential cytoplasmic contamination and cell lysis via dot blot analyses detecting RpoA, the α-subunit of the RNA-Polymerase (for details see section “Dot Blot Analysis”). A representative dot blot is shown in Supplementary Figure S4B, indicating equally low levels of cytoplasmic contamination for all periplasmic fractions.

As *vps* expression was unaltered in the Δ*pglL*_Vc_Δ*rbmD* mutant, we hypothesized that the alterations in biofilm amounts and architecture could be due to differential secretion of the T2SS substrates RbmA, RbmC or Bap1, which represent three major adhesive protein components of the *V. cholerae* biofilm matrix (Absalon et al., 2011; Berk et al., 2012). We therefore deleted *rbmA*, *rbmC* and *bap1* independently in the WT, Δ*pglL*_Vc_, Δ*rbmD*, and Δ*pglL*_Vc_Δ*rbmD* backgrounds and tested biofilm formation. Individual mutants Δ*rbmA*, Δ*rbmC*, and Δ*bap1* exhibit similar biofilm levels compared to the WT in the static biofilm assay (Figure 7A), which is concordant with a previous report as single deletion of each of the three matrix proteins does not impact biofilm formation (Fong et al., 2006). The absence of PglL_Vc_ and RbmD still resulted in increased biofilm formation in the Δ*rbmC* and Δ*bap1* backgrounds (Figure 7A). Similarly, Δ*pglL*_Vc_ and Δ*rbmA*Δ*pglL*_Vc_ showed equally enhanced biofilm levels compared to the WT. Deletion of *rbmA* in the Δ*rbmD* or Δ*pglL*_Vc_Δ*rbmD* background negated the RbmD-dependent biofilm phenotypes and resulted in significant lower biofilm amounts compared to the parental Δ*rbmD* or Δ*pglL*_Vc_Δ*rbmD* strains (Figure 7A). This phenotypic analysis thus revealed synthetic genetic interactions between *rbmA* and *rbmD*.

To further investigate the relationships between RbmD and RbmA, we examined RbmA trafficking using strains expressing a His-tagged derivative allowing immunoblot analysis. In the absence of PglL_Vc_ and RbmD, RbmA was relatively efficiently secreted into the supernatant with a reciprocal minority localized in the periplasmic fraction. Remarkably, expression of solely RbmD, but not PglL_Vc_, effectively reversed RbmA localization resulting in its relative accumulation in the periplasmic fraction and reduced translocation into the supernatant (Figure 7B). RbmD thus diminishes the secretion efficacy of RbmA, providing an example for differential secretion of a T2SS substrate modulated by the status of RbmD.

Having revealed a RbmD-dependent periplasmic localization of RbmA, we tried to pinpoint the origin of the glycan potentially transferred by RbmD. Based on the differential secretion pattern of CT in presence or absence of the VPS, we focused on a strain lacking the *vps-*I gene cluster. In the Δ*vps-*I background the RbmD-dependent periplasmic accumulation of His-tagged RbmA was negated and a robust translocation of His-tagged RbmA was observed (Figure 7C). Concordant to the CT data, RbmD-dependent periplasmic accumulation of His-tagged RbmA was still observed in a Δ*rfb* background (Figure 7D). This suggests that the *vps* cluster can provide a glycan precursor transferred by RbmD.

## Discussion

Here, we establish a clear association between *O*-linked protein glycosylation mediated by the oligosaccharyltransferase PglL_Vc_ and the efficacy of type II protein secretion-dependent processes in the human pathogen *V. cholerae*. These findings define a first connection between protein glycosylation and processes critical to *V. cholerae* fitness in and outside of its host, such as differential secretion of T2SS-dependent substrates including CT, chitinases and the biofilm matrix protein RbmA. Moreover, we show that mutants lacking RbmD, whose functions remain unknown but shares structural features with PglL_Vc_, exhibit phenotypic perturbations analogous to those lacking PglL_Vc_.

These findings raise the obvious question as to how *O*-linked protein glycosylation status mediated by PglL_Vc_ might impact on the translocation of type II protein secretion substrates across the outer membrane. Perhaps the simplest explanation would be that one or more of the glycoproteins targeted by PglL_Vc_ influences type II protein secretion proficiency and that the activity or functionality of that target protein is influenced by its glycosylation status. Here, obvious candidates for such glycoproteins would be DegP as well as other periplasmic chaperones such as SurA and FkpA acting alone or in concert. Nonetheless, we hypothesize that glycosylation of these and/or other proteins might diminish their intrinsic functionality or abundance. In this model, lack of glycosylation would thus enhance their activity or levels to foster the terminal steps of the T2SS. Confirmation of this model requires a more thorough characterization of the *V. cholerae* glycoproteome and the effects of *O*-linked protein glycosylation on glycoprotein abundance and functionality. Notably, *pglL_*V*__*c*_* is located upstream of the *msh*-operon encoding a surface pilus termed the mannose-sensitive hemagglutinin (MSHA). Although not identified along our study, it is tempting to speculate that *O*-glycosylation also targets components of MSHA or affect their secretion as *O*-glycosylation of pili components has been demonstrated for other bacteria (Aas et al., 2007; Egge-Jacobsen et al., 2011). Notably, MSHA is important for the first steps in biofilm formation by facilitating adherence to abiotic and chitinous surfaces as well as for the pharyngeal colonization of nematodes (Watnick et al., 1999; Chiavelli et al., 2001; Meibom et al., 2004; List et al., 2018). Thus, it cannot be excluded that the biofilm phenotypes of *pglL*_Vc_ and *rbmD* mutants observed herein are at least partially associated to differential MSHA functionality.

An intriguing aspect of this work relates to role of RbmD whose mutation phenocopies that of the *pglL*_Vc_ null mutant. To our knowledge, this is one of the first reports of these phenotypes associated with the failure to express this protein in *V. cholerae* WT. Notably, *V. cholerae* can undergo phenotypic variation generating smooth and rugose variants. The later shows elevated VPS-production, increased biofilm formation and enhanced resistance to several environmental stresses. While the work herein is based on a smooth *V. cholerae* isolate, Fong and Yildiz (2007) analyzed biofilm formation of a *rbmD*-mutant in a rugose variant. In the rugose background deletion of *rbmD* resulted in less compact biofilms and larger cell aggregates compared to the parental rugose variant. However, direct comparison of the results from our study and the work by Fong et al. is difficult, due to the differential phenotypic background and different *V. cholerae* isolates used. Based on the results presented herein, we surmise that RbmD impacts on the same pathway as that modulated by PglL_Vc_ in a non-redundant fashion. Besides similarity PglL_Vc_, RbmD is also related to WaaL-type O-antigen ligases and members of the SEDS (shape, elongation, division and sporulation) protein family members that serve as bacterial cell wall polymerases (Meeske et al., 2016). The potential for RbmD to have a role other than in protein glycosylation is intriguing especially given its inability to use the UndPP-diNACBac donor that can be used by virtually all *O*-OTases tested to date.

Another obvious point of interest relates to the nature of the endogenous glycan used by PglL_Vc_ and potentially RbmD. Computational analyses of the Sequence Similarity DataBase revealed no obvious similarities of the entire gonococcal *pgl* gene cluster in *V. cholerae.* However, similarity searches for orthologs to the individual gonococcal *pgl* genes revealed several hits in two gene clusters (VC0240–VC0263 and VC917–VC927) encoding enzymes for the O-antigen biosynthesis and the *Vibrio* exopolysacharide (VPS), respectively. Deletion of the *vps*-I gene cluster (but not that for the O-antigen biosynthesis) phenocopied *pglL*_Vc_ and *rbmD* mutants in the enhanced secretion of both CT and RbmA, providing first evidence that the diverse sugars present in the VPS [i.e., α-GulNAcAGly, α-Glc*p*, β-Glc*p*, α-Gal*p*, and α-D-GlcNAc (Yildiz et al., 2014)] or combinations thereof may serve as at least one source for the transferred *O*-glycan in *V. cholerae*. Notably, the VPS oligosaccharide is notoriously recalcitrant to biochemical characterization as it is bound to a yet unidentified component, which gives it high viscosity and which would theoretically complicate its structural characterization by MS as a post-translational protein modification.

Further insights into the mechanisms operating here will undoubtedly require elucidation of the cognate glycoform utilized by PglL_Vc_ and potentially by RbmD. Along this line we tried to identify the glycan present in *V. cholerae*. Briefly, His-tagged DegP was isolated in absence and presence of PglL_Vc_ and/or RbmD and subjected to MS analysis, but no defined modification could be identified. It should be emphasized that detection and identification of a glycan with unknown composition is not straightforward and requires substantial expertise and instrumentation. A series of unfavorable limitations decreased the likelihood of glycan identification: (i) Overexpression of periplasmic His-tagged DegP reduced fitness of *V. cholerae*. Thus, the yield of purified protein was relatively low. (ii) The chemical composition of the natural glycan is not only unknown, but might exhibit heterogeneity. Thus, the glycosylated population may not represent a single peak in the MS. (iii) *O*-glycosylation might render protein stability. (iv) Based on our results using the gonoccocal glycan only a minor population of substrate is glycosylated, e.g., ∼1% in case of DegP in presence of the diNAcBac glycan and PglL_Vc_. It should be noted, that such low modification rates could massively affect overall DegP chaperone activity in the periplasm as DegP monomers assemble from trimers and hexamers to multimeric polyhedral cages with up to 24 subunits (Krojer et al., 2002; Kim and Sauer, 2014). However, such modification rates further reduce the chances for identification of the natural glycan. Based on these limitations the identification of the cognate glycan seems to be a challenging task for the future.

In summary, we herein reveal multiple gene interactions that ultimately impact on the efficacy of the *V. cholerae* T2SS at the level of translocation of substrates from the periplasm to the extracellular milieu. T2SS modulation affects several stages of the pathogens’ lifecycle as highlighted by the diverse phenotypes reported herein. It is tempting to speculate that *O*-glycosylation provides a post-translational mechanism allowing bacterial cells to accumulate effector proteins, like RbmA, chitinases or CT in the periplasm to be readily available for release when the cells settle down on the new substratum for biofilm formation or at the colonization site in the gastrointestinal tract. Thus, *O*-glycosylation via PglL_Vc_ and possibly RbmD could represent a fine-tuned feedback mechanism controlling release of T2SS effectors by modulation of secretion efficacy.

## Data Availability Statement

All datasets generated for this study are included in the article/Supplementary Material.

## Author Contributions

DV, FM, KP, JA, MF, JR, MK, and SS designed the study. DV, FM, KP, DL, JA, LL, L-MM, TK, MT, AS, FZ, RB-G, JR, MK, and SS performed the experiments and analysis. DV, FM, KP, JA, FZ, MF, JR, MK, and SS contributed to the discussion and data evaluation. DV, FM, JR, MK, and SS wrote the manuscript.

## Conflict of Interest

The authors declare that the research was conducted in the absence of any commercial or financial relationships that could be construed as a potential conflict of interest.

## Footnotes

1 http://www.comstat.dk
