# Supplementary material for: A Broad Spectrum Protein Glycosylation System Influences Type II Protein Secretion and Associated Phenotypes in Vibrio cholerae
Source: Front Microbiol. 2019 Dec 3;10:2780. doi: 10.3389/fmicb.2019.02780 (PMC6901666; doi:10.3389/fmicb.2019.02780)
Supplement: Supplementary file 1 [file Data_Sheet_1.DOCX]

**Supplementary Material**

**
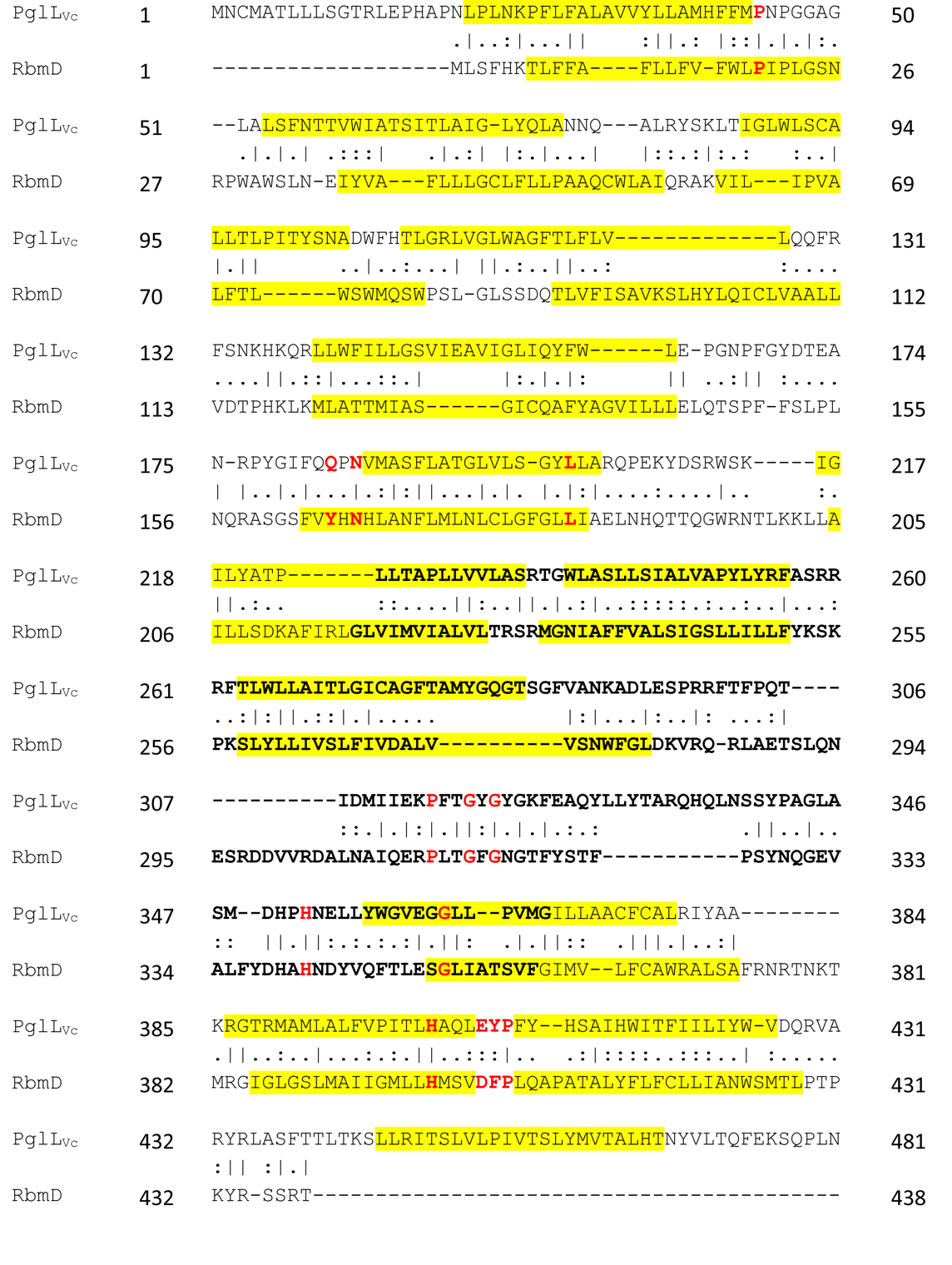
**

**Supplemental Figure S1. Conserved amino acid residues and transmembrane domain topology in the putative *O*-OTases of *V. cholerae*.** Sequence alignment reveals that PglL_Vc_ and RbmD share only 17% identity and 32% similarity on the protein level, but relevant amino acids reported to be required for optimal function of *O*-OTases from *Neisseria meningitidis*, *Burkholderia thailandensis, Acinetobacter baylyi* and *Ralstonia pickettii* are conserved (indicated in red) (Musumeci et al., 2014). In *N. meningitidis* H349 (equivalent of H352 of PglL_Vc_ or H341 of RbmD) was determined as essential for interaction with lipid-linked glycan (Musumeci et al., 2014). For the alignment, Pairwise Sequence Alignment - EMBOSS Needle tool was used. Wzy_C signitaure domain is highlighted in bold (Kanehisa and Goto, 2000) and transmembrane domains predicted by TMHMM are indicated in yellow (TMHMM).

Kanehisa, M., and Goto, S. (2000). KEGG: kyoto encyclopedia of genes and genomes. *Nucleic Acids Res* 28(1)**,** 27-30.

Musumeci, M.A., Faridmoayer, A., Watanabe, Y., and Feldman, M.F. (2014). Evaluating the role of conserved amino acids in bacterial *O*-oligosaccharyltransferases by in vivo, in vitro and limited proteolysis assays. *Glycobiology* 24(1)**,** 39-50. doi: 10.1093/glycob/cwt087.

TMHMM, C.f.b.s.a.: Tehnical University of Denmark. Available: <http://www.cbs.dtu.dk/services/TMHMM/> [Accessed].

**
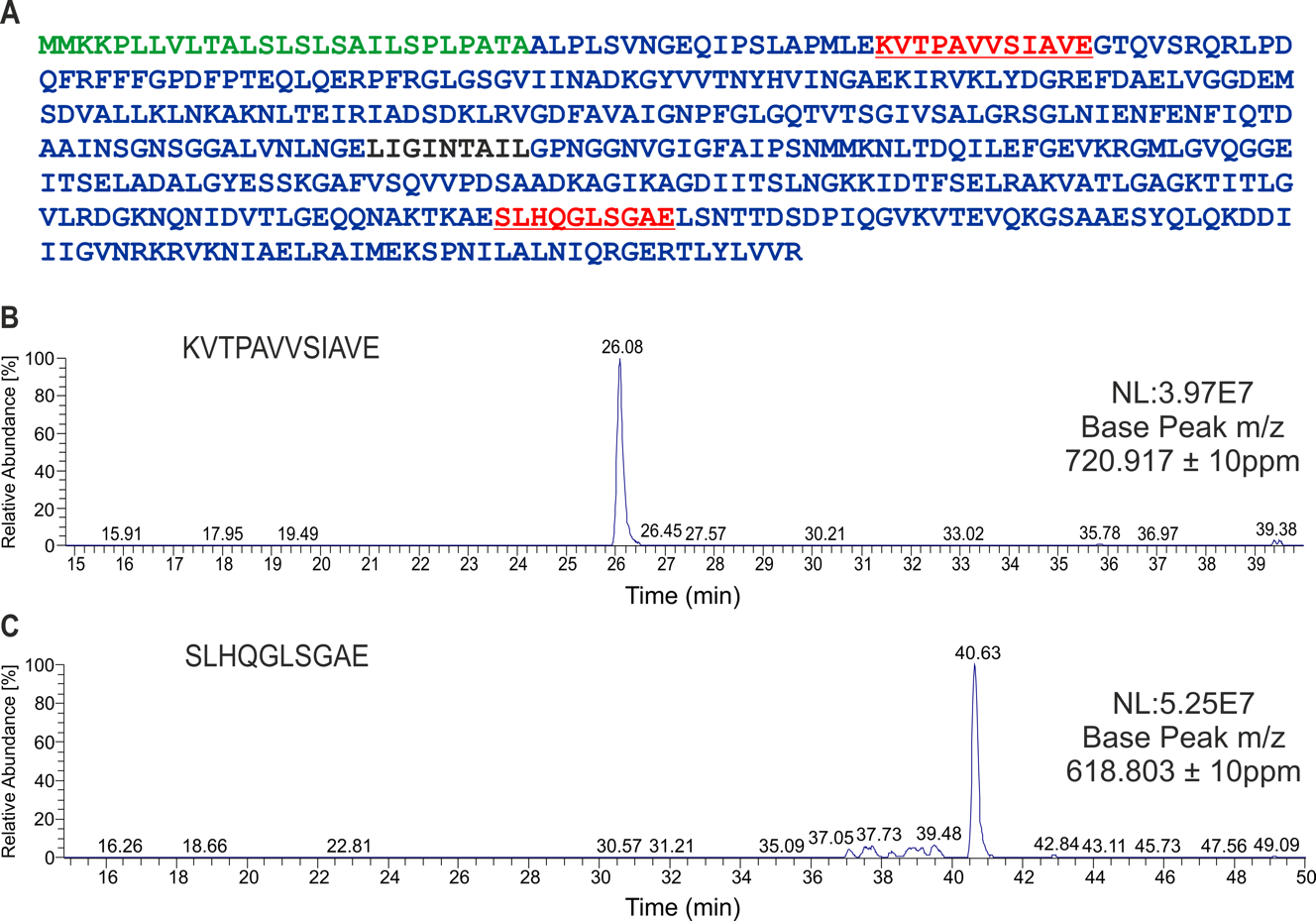
**

**Supplemental Figure S2. LC-MS/MS analyses of DegP glycosylation. (A)** A total of 98% sequence coverage of DegP was achieved from trypsin and GluC in-gel digest of DegP. Blue areas represent amino acid sequences detected from trypsin and GluC digested samples. Underline, bold and red sequences represent the smallest common glycosylated sequence identified. Signal peptide is indicated in green. **(B and C)** Xtracted Ion Chromatograms (XIC) from in-gel GluC digest of precursor ion masses of DegP modified peptides centered on the accurate theoretical monoisotopic masses with a 10 ppm mass window. **(B)** XIC of the theoretical doubly charged monoisotopic mass of the modified peptide ^47^KVTPAVVSIAVE^58^. Only a single peak was detected in the chromatogram indicating that only a single modified form of the peptide is present in the sample. **(C)** XIC of the theoretical doubly charged monoisotopic mass of the modified peptide ^371^SLHQGLSGAE^380^. Only a single peak was detected in the chromatogram indicating that only a single modified form of the peptide is present in the sample.

**
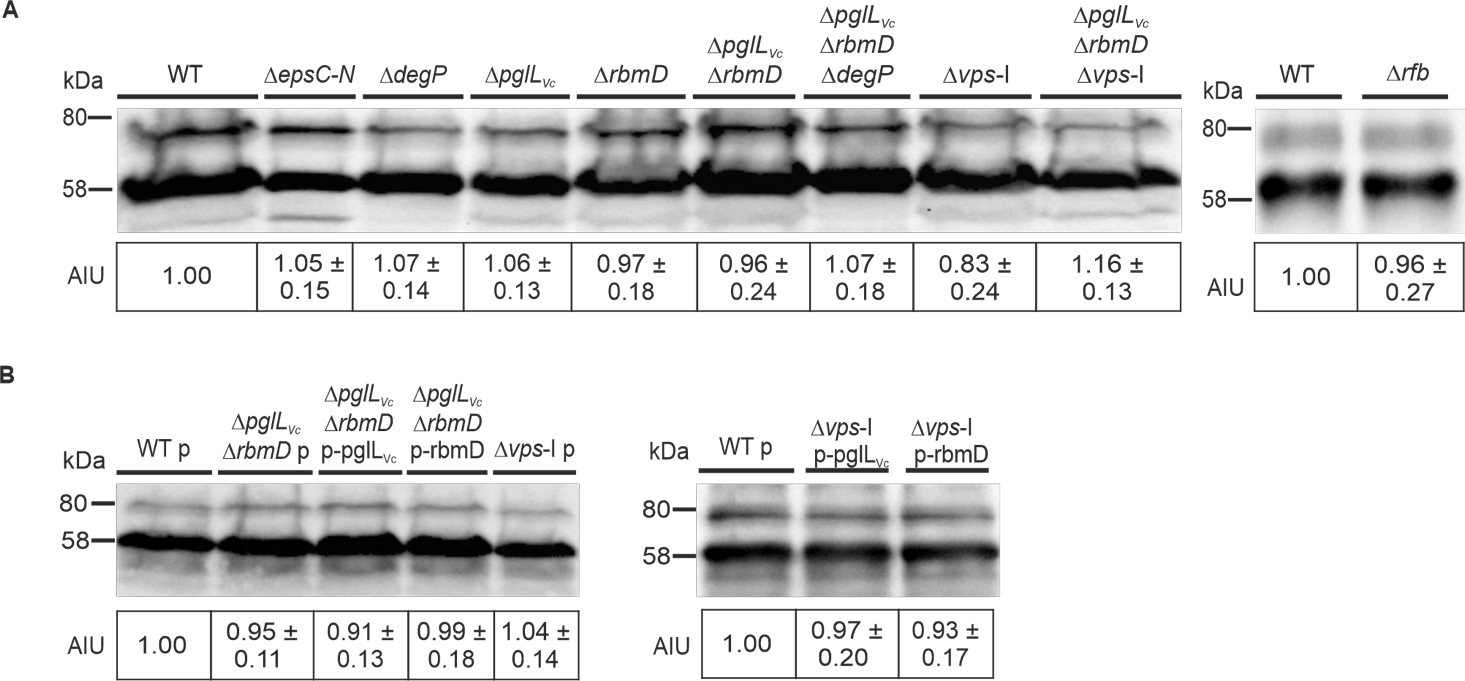
**

**Supplemental Figure S3. Equal CT expression levels was confirmed by immunoblot analyses for all strains analyzed for CT secretion by ELISA.** **(A and B)** The amount of CT was determined in whole cell extracts of respective strains by immunoblot analyses. All strains were grown under virulence gene factor expressing conditions (AKI conditions) to induce CT expression. Shown are representative immunoblots detecting the abundant CT-B pentamer at approximately 55 kDa (serving as controls for Figure 3). Lines to the left indicate the molecular masses of the protein standards in kDa. Semiquantitative densitometric evaluation was performed with the Quantity One software (Bio-Rad Laboratories) and is indicated below the immunoblot as arbitrary intensity units (AIU) normalized to WT (A) or WT with empty vector (WT p, B), which was always set to 1. The data is given as mean with standard deviation (n ≥ 5).

**
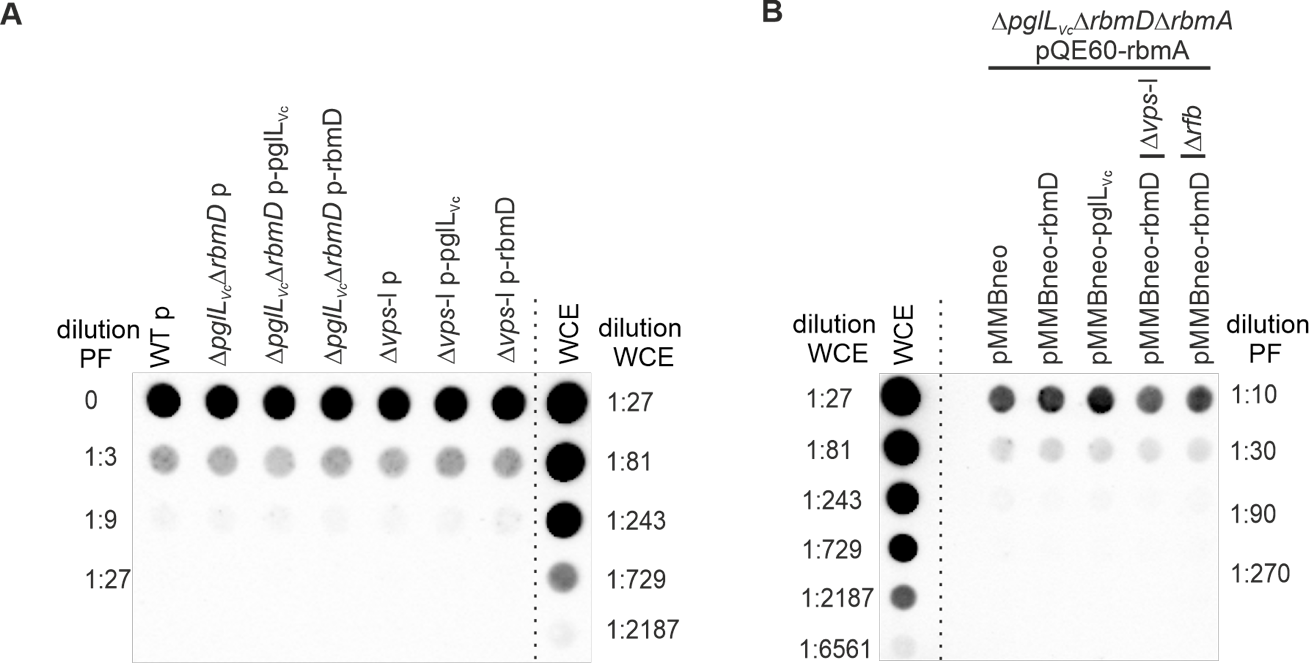
**

**Supplemental Figure S4. Quality control of periplasmic fractions (PF) via dot blot analyses detecting RpoA.** Shown are representative dot blots detecting cytoplasmic RpoA, the α-subunit of the RNA-Polymerase, in periplasmic fractions (PF) of the indicated strains (serving as controls for PF samples used in Figure 3 and 7). **(A)** Equal proteins amounts (Bradford) of PF or dilutions thereof (3-fold as indicated) were spotted along with appropriate dilutions (3-fold as indicated) of whole cell extracts (WCE) derived from WT. **(B)** TCA-precipitated PF were adjusted to equal protein amounts (Bradford) and appropriate dilutions (3-fold as indicated) were spotted along with appropriate dilutions (3-fold as indicated) of whole cell extracts (WCE) derived from WT on a nitrocellulose membrane and subjected to RpoA detection.

**
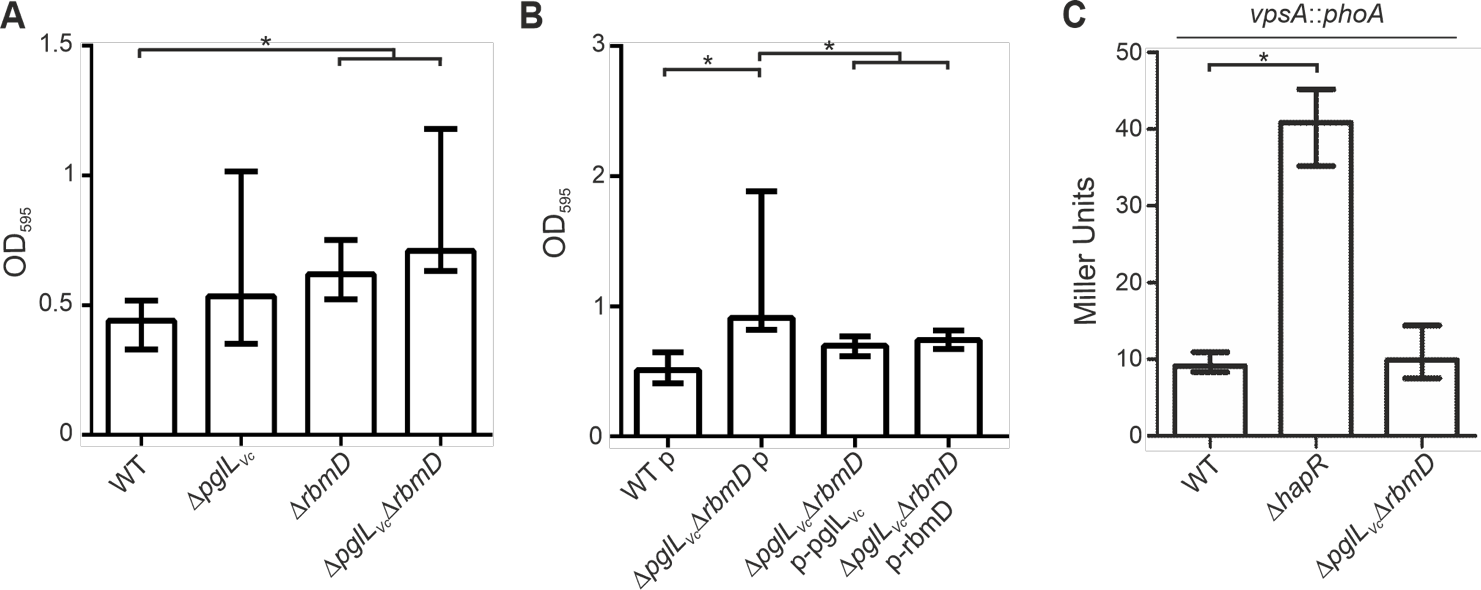
**

**Supplemental Figure S5. Extended analysis of *O*-OTase dependent biofilm phenotypes and impact on *vps* expression. (A and B)** The biofilm formation capacity of the strains indicated on the x-axis was assayed under static conditions by crystal violet staining and subsequent determination of the OD_595_ (n ≥ 24). **(A)** Biofilms of WT, Δ*pglL_Vc_*, Δ*rbmD* and Δ*pglL_Vc_*Δ*rbmD* were quantified after 24 h. **(B)** *In trans* expression of PglL_Vc_ or RbmD partially restores biofilm to WT levels at 24 h. **(C)** Alkaline phosphatase activities (in Miller Units) were measured from ON cultures of the WT, ∆*hapR* and ∆*pglL_Vc_*∆*rbmD* with a chromosomal *vpsA-phoA* transcriptional fusion (n ≥ 10). **(A to C)** Data is presented as median with interquartile range. Asterisks highlight significant differences between respective data sets (* *P* < 0.05 Kruskal-Wallis test followed by *post hoc* Dunn’s multiple comparisons).


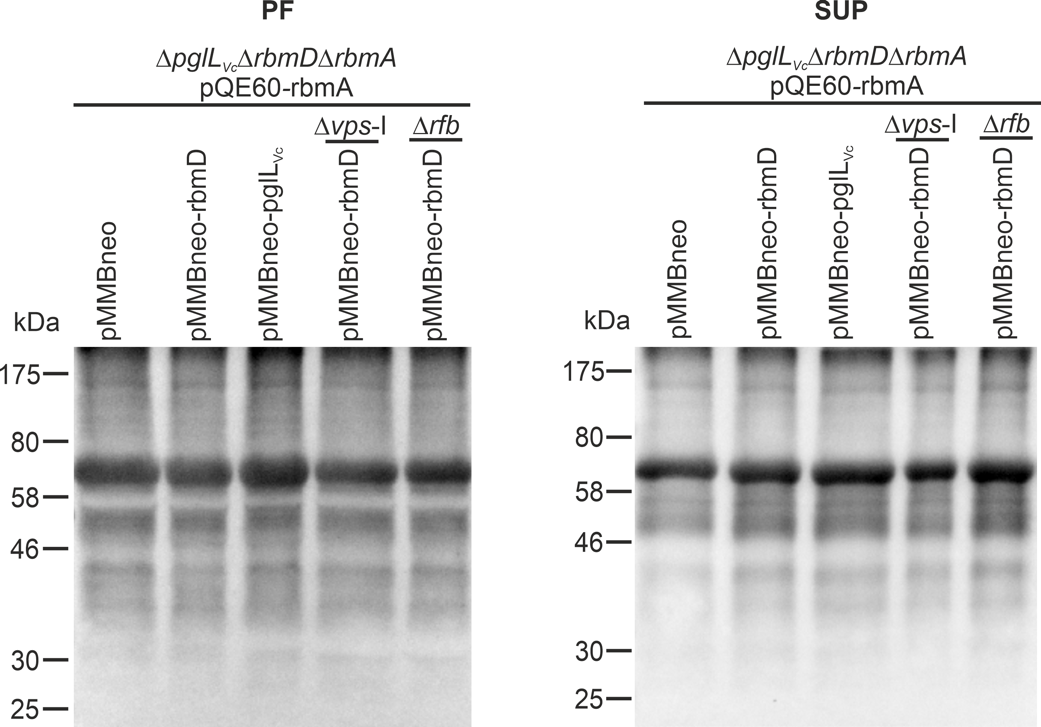


**Supplemental Figure S6. Immunoblot controls detecting BSA in TCA-precipitated periplasmic fractions and culture supernatants.** Shown are representative immunoblots detecting BSA (66.5 kDa) in periplasmic fractions (PF) and supernatants (SUP) of the indicated strains. PF and supernatants were spiked with BSA (0.1 µg/ml) prior TCA-precipitation and precipitated samples were then analyzed by immunoblot for equal BSA amounts to exclude differential efficiency in protein precipitation (serving as controls for Figure 7). Equal amounts of proteins for PF or SUP were loaded according to Bradford to allow direct comparison of the fractions.
